# Single-cell transcriptomics reveal a unique memory-like NK cell subset that accumulates with ageing and correlates with disease severity in COVID-19

**DOI:** 10.1186/s13073-022-01049-3

**Published:** 2022-05-03

**Authors:** Chuang Guo, Mingming Wu, Beibei Huang, Rui Zhao, Linlin Jin, Binqing Fu, Ping Wang, Dongyao Wang, Meijuan Zheng, Jingwen Fang, Haiming Wei, Kun Qu, Fang Ni

**Affiliations:** 1 Department of Hematology, The First Affiliated Hospital of USTC, The CAS Key Laboratory of Innate Immunity and Chronic Disease, School of Basic Medical Sciences, Division of Life Sciences and Medicine, University of Science and Technology of China, Hefei, China; 2 Department of Oncology, The First Affiliated Hospital of USTC, Division of Life Sciences and Medicine, University of Science and Technology of China, Hefei, China; 3 Institute of Immunology, University of Science and Technology of China, Hefei, China; 4 Department of Clinical Laboratory, The First Affiliated Hospital of Anhui Medical University, Hefei, China

**Keywords:** Ageing, NK cell, Single-cell RNA-seq, Type-I interferon, COVID-19

## Abstract

**Background:**

Natural killer (NK) cells are innate lymphoid cells that mediate antitumour and antiviral responses. However, very little is known about how ageing influences human NK cells, especially at the single-cell level.

**Methods:**

We applied single-cell sequencing (scRNA-seq) to human lymphocytes and NK cells from 4 young and 4 elderly individuals and then analysed the transcriptome data using Seurat. We detected the proportion and phenotype of NK cell subsets in peripheral blood samples from a total of 62 young and 52 elderly healthy donors by flow cytometry. We also used flow cytometry to examine the effector functions of NK cell subsets upon IFN-α/IL-12+IL-15/K562/IL-2 stimulation in vitro in peripheral blood samples from a total of 64 young and 63 elderly healthy donors. We finally studied and integrated single-cell transcriptomes of NK cells from 15 young and 41 elderly COVID-19 patients with those from 12 young and 6 elderly healthy control individuals to investigate the impacts of ageing on NK cell subsets in COVID-19 disease.

**Results:**

We discovered a memory-like NK subpopulation (NK2) exhibiting the largest distribution change between elderly and young individuals among lymphocytes. Notably, we discovered a unique NK subset that was predominantly CD52^+^ NK2 cells (NK2.1). These memory-like NK2.1 cells accumulated with age, exhibited proinflammatory characteristics, and displayed a type I interferon response state. Integrative analyses of a large-cohort COVID-19 dataset and our datasets revealed that NK2.1 cells from elderly COVID-19 patients are enriched for type I interferon signalling, which is positively correlated with disease severity in COVID-19.

**Conclusions:**

We identified a unique memory-like NK cell subset that accumulates with ageing and correlates with disease severity in COVID-19. Our results identify memory-like NK2.1 cells as a potential target for developing immunotherapies for infectious diseases and for addressing age-related dysfunctions of the immune system.

**Supplementary Information:**

The online version contains supplementary material available at 10.1186/s13073-022-01049-3.

## Background

Human ageing is a complex, dynamic process that ultimately leads to increased susceptibility to multiple chronic diseases, disability, and death [1]. With age, the immune system undergoes dramatic changes, which continuously progress to a state called immunosenescence [2]. An interesting phenomenon associated with immunosenescence is inflammaging, which is characterized by chronic low-grade inflammation with elevated levels of several proinflammatory cytokines, such as IL-6, IL-1, interferon-γ (IFN-γ), tumour necrosis factor (TNF), and diverse chemokines [3]. Inflammaging is considered a central hallmark of human ageing [4, 5], but its influence on specific types of immune cells remains largely unexplored.

NK cells are part of the innate immune system and function to eliminate infected or transformed cells, and NK cells also function as mediators of adaptive immunity [6, 7]. Following stimulation with viruses or cytokines, human NK cells can acquire adaptive or memory-like properties, including long-term persistence and enhanced functional responsiveness, similar to adaptive memory T cells [8–14]. In humans, NK cells are typically subdivided into cytotoxic CD56^dim^ NK cells and CD56^bright^ NK cells, which are less cytotoxic but produce larger amounts of cytokines, including IFN-γ, TNF-α, GM-CSF, and IL-10 [15]. With ageing, there is a reduction in CD56^bright^ NK cells that is accompanied by an expansion of CD56^dim^ NK cells; together, these changes result in an overall increase in the absolute number of NK cells [16–19]. However, it should be noted that age-related changes in human NK cell functionality have been inconsistent and controversial. For instance, studies have demonstrated reduced, normal, or even increased IFN-γ production upon NK cell activation in older adults [20, 21]. Although efforts have been made to investigate age-related changes in NK cells, such studies have been somewhat limited by the technical approaches. High-resolution and unbiased analyses of the impacts of ageing on human NK cells are needed, especially within the conceptual framework of the traditional CD56^dim^ NK cells and CD56^bright^ NK cells.

Single-cell RNA sequencing (scRNA-seq) provides an unbiased method to decipher cellular heterogeneity and cell states based on the transcriptomes of individual cells [22]. Here, we performed scRNA-seq and flow cytometry analyses, together with in vitro functional assays, to characterize age-associated alterations in human NK cells. We discovered a subpopulation of NK2 cells that seemed to be phenotypically memory-like NK cells and exhibited the largest distribution change between elderly and young individuals among 9 blood immune cell subpopulations. We further discovered a unique NK subset that was predominantly CD52^+^ NK2 cells in elderly individuals. These memory-like CD52^+^ NK2 cells (herein termed NK2.1 cells) accumulated with age and exhibited proinflammatory characteristics. NK2.1 cells in elderly individuals displayed elevated sensitivity to type I interferon stimulation in vitro. Finally, integrative analyses of a large-cohort COVID-19 single-cell transcriptomic dataset with our single-cell datasets revealed that NK2.1 cells from elderly COVID-19 patients are enriched for genes related to type I interferon signalling (e.g. *ISG15*, *ISG20*), which is positively correlated with disease severity in COVID-19. It therefore appears that this memory-like NK2.1 subset represents a potential target for immunotherapies to treat infectious diseases and should be considered during the development of immunological interventions for older adults.

## Methods

### Study design

We used scRNA-seq technology to capture the transcriptomes of human NK cells and assess how ageing influences human NK cells. We applied flow cytometry to confirm observations from sequencing data. We also performed an integrative analysis of COVID-19 single-cell transcriptomes of NK cells with those from healthy control individuals to investigate the impacts of ageing on NK cell subsets in COVID-19 disease. Details on human sample collection and data processing are described below.

### Human samples

All healthy blood samples were de-identified. All blood samples for scRNA-seq and flow cytometry experiments were obtained from young and elderly healthy donors who came to the Health Management Center of the First Affiliated Hospital of Anhui Medical University (Hefei, China) for physical examinations; none of the donors had a history of cancer, HBV, HCV, or HIV infection; autoimmune disease; diabetes; hypertension; or steroid usage. Clinical characteristics are listed in Additional file 1: Table S1. All experiments using human blood samples in this study were approved by the Ethics Committee of the University of Science and Technology of China (approval no. 2020-KY196). Informed consent was obtained from all donors. Fresh whole blood samples were collected in 2-mL tubes containing ethylenediaminetetraacetic acid (EDTA). PBMCs were isolated from whole blood within 4 h of sample collection with Ficoll-Paque gradient centrifugation (TBDsciences) according to the manufacturer’s instructions.

In this study, we performed scRNA-seq on PBMCs and purified NK cells from 4 elderly and 4 young healthy individuals. We applied flow cytometry in peripheral blood samples (i) from 62 young and 52 elderly healthy donors to detect the proportion and phenotype of NK cell subsets, (ii) from 26 young and 27 elderly healthy donors for IFN-α stimulation experiments, (iii) from 18 young and 16 elderly healthy donors for IL-12/IL-15 stimulation experiments, (iv) from 10 young and 10 elderly healthy donors for K562 stimulation experiments, and (v) from 10 young and 10 elderly healthy donors for IL-2 stimulation experiments. Details on the sample information of healthy donors are provided in Additional file 1: Table S1.

### Detection of CMV serostatus

Antibodies to HCMV were tested with ELISA using commercial kits for IgM (Captia™ Cytomegalovirus IgM, Trinity biotech) and IgG (Captia™ Cytomegalovirus IgG, Trinity biotech). The CMV serostatus of donors used in this study is shown in Additional file 1: Table S1.

### In vitro stimulation of NK cells

Freshly isolated PBMCs from healthy individuals were cultured for 16 h in RPMI 1640 medium containing 20% foetal bovine serum (Sigma) in the presence of the following stimuli: 1000 U/mL recombinant human IFN-α (Biolegend) or 10 ng/mL recombinant human IL-12 (Peprotech) plus 100 ng/mL IL-15 (Peprotech). GolgiStop (Sigma) was added to the medium for 4 h for stimulation, and antihuman CD107a antibody (BD) was added for 2 h for stimulation. Cells were cultured in a medium alone as a negative control.

### Collection of COVID-19 samples and selection of NK cells

Previously published scRNA-seq datasets from Ren et al. [23] were downloaded from the Gene Expression Omnibus (GEO) database (accession number GSE158055); processed datasets from Schulte-Schrepping et al. [24] (Bonn data and Berlin data) were downloaded from FastGenomics (https://www.fastgenomics.org) as Seurat objects (https://beta.fastgenomics.org/p/Kraemer_2021_COVID19_NK), and datasets from Witkowski et al. [25] were downloaded from the GEO database (accession number GSE184329).

NK cells present in each dataset were selected in a three-step process: (i) we extracted PBMC-derived single-cell sequencing datasets based on the sample source, (ii) we extracted the qualified samples (young ≤ 30 years; elderly ≥ 65 years) based on the age information, and (iii) NK cells were selected based on the cell type label provided by Schulte-Schrepping et al.’s study [24]. We further checked the selected NK cells using classical NK cell markers (*KLRF1*, *GNLY*, *NKG7*) and applied the SingleR [26], which is widely used for cell type identification, to remove non-NK cells.

### Single-cell RNA sequencing

For the scRNA-seq analysis of human blood lymphocytes and NK cells, lymphocytes were sorted as CD45^+^ cells, and NK cells were sorted as CD3/CD19/CD20/CD14^−^CD7^+^ cells using FACS (SONY SH800S); the purity was above 95%. The cells from 4 elderly individuals were pooled in one tube, and the cells from 4 young individuals were pooled in another tube. We then counted and resuspended the pooled cells at a concentration of 1000 cells/μL, aiming for an estimated 6000 cells per library, following the instructions of the single-cell 3′ solution v3 reagent kit (10X Genomics). Single-cell libraries were constructed strictly according to the manufacturer’s standard protocols. Each sequencing library was generated with a unique sample index. Libraries were sequenced on the Illumina NovaSeq 6000 system.

### RNA sequencing data processing (QC and dimensionality reduction)

Following sequencing data acquisition, the FASTQ files of each human sample were aligned to the hg19 reference genome, and UMI counts were quantified using the 10X Genomics Cell Ranger pipeline (v3.1.0, 10X Genomics [27]) with default parameters. Quality control and dimensionality reduction were performed in R v3.6.3 using the Seurat package v3.2.2 [28]. For the initial QC step, we created Seurat objects for the young and elderly groups and filtered out the cells that expressed < 400 genes, > 3000 genes or > 10% mitochondrial genes. Moreover, genes expressed in fewer than 5 cells were excluded. Next, we used the “IntegrateData” function to integrate the top X (parameter for CCA) dimensions of the two objects for the anchor weighting procedure. Then, we selected the top Y (parameter for PCA) PCA dimensions to perform PCA on the integrated object and set the resolution as Z (parameter for resolution) to obtain the clusters (parameter setting: CD45^+^ lymphocyte clustering: CCA = 20, PCA = 25, resolution = 0.29; Lin^−^CD7^+^ NK cell clustering: CCA = 25, PCA = 25, resolution = 0.5; NK cell clustering from COVID-19 and HD samples: CCA = 25, PCA = 25, resolution = 0.5). We removed the clusters that contained fewer than 2% of the cells after clustering. Using SingleR [26], a small number (469 cells) of contaminated non-NK cells was found in the COVID-19 source dataset, which was excluded from further analysis. To quantify the similarity of the two datasets, we calculated the Jaccard index of subsets using the top 50 ranking cluster-specific marker genes.

### Normalization and differential expression analysis

The gene expression count matrix for each cell was normalized using the “pp.normalize_total” function of Scanpy [29] and log-transformed to the product (base e) after the addition of a pseudocount of 1. We performed differential gene expression analysis for our identified cell subpopulations using the Wilcoxon rank-sum test within the Seurat “FindAllMarkers” function. We also performed differential gene expression analysis between young and elderly individuals using the “FindMarkers” function with multiple default thresholds in the Seurat package. For Gene Ontology (GO) analysis of DEGs, we then uploaded these DEG groups to the Metascape [30] website (https://metascape.org/gp/index.html#/main/step1) and used the default parameters to perform Gene Ontology (GO) analysis of the gene list.

### Standardization of gene set expression

We downloaded the gene set (such as the top 50 age-associated genes obtained from Peters et al. [31]) and calculated the signature score to compare the enrichment of the gene set in the cells or samples. For the normalized expression data, we first summed the *Z*-score of the expression value of each gene in the gene set in all cells and then normalized them to values from 0 to 1 between cells as a signature score. *P*-values were obtained with the Wilcoxon rank-sum tests.

### Evaluating the proportion of 9 lymphocyte subpopulations from bulk RNA-seq data

We first identified the expression of signature genes of each cell subpopulation in our single-cell analysis (Additional file 1: Table S2) and then calculated the fold change of each signature gene (elderly vs. young) in downloaded bulk RNA-seq datasets of PBMCs from 10 young and 10 elderly individuals [32]. We used the average fold change of all signature genes to estimate the enrichment of each cell subpopulation in elderly individuals. *P*-values were estimated by two-sided Student’s *t*-tests.

### Estimating the connectivity of NK subsets using partition-based graphical abstraction (PAGA)

For similarity-based cell network analysis and visualization, we used tools from the Python (v3.6) library Scanpy (v1.5.1). The input matrix for Scanpy is the normalized and log-transformed highly variable 2200 gene expression data. PAGA [33], which is a high-resolution pseudotime prediction algorithm, was then used to estimate and quantify the connectivity of partitions (the NK1.1, NK1.2, NK2.1, NK2.2, and NK2.3 cell subsets) of the single-cell graph. For the PAGA graph, the node size is proportional to the number of cells in the subset, and the thickness of the edge shows the strength of confidence in the connectivity of partitions between subsets (parameters: n_pcs = 25, n_neighbours = 5, random_state = 3).

### Transcriptional regulon enrichment analysis

We used SCENIC [34] (version 1.1.2.2) to predict TF activities from the scRNA-seq data. The normalized and log-transformed variable gene expression matrix of NK cells was used as the input feature for SCENIC. First, TF-gene coexpression modules were defined in a data-driven manner with GENIE3 [34, 35]. Then, those modules are refined via RcisTarget [34] by keeping only those genes that contain the respective TF binding motif. AUCell [34] scores individual cells by assessing for each TF separately whether target genes are enriched in the top quantile of the cell signature. For the regulons determined by SCENIC, we retained regulons with activity differences greater than 0.03 between the young and elderly individuals in each subset.

### Using PAGA to order cells in pseudotime along a trajectory

PAGA-initialized single-cell embedding for selected cell types was estimated with Scanpy v1.5.1 on the normalized and log-transformed expression matrix, with the following parameters: n_pcs = 20, n_neighbors = 30, and random_state = 3. The differentiation map was estimated using PAGA [33] implemented in Scanpy v1.5.1 with the same parameters. Twenty diffusion components are used as input to generate the ForceAtlas2 layout. As NK1.1 cells showed enrichment of gene expression for the CD56bright NK cell signature genes, we randomly selected a NK1.1 cell as the root cell to construct the diffusion pseudotime on the selected NK cell types. We used the neighbours() function and the dpt() function with a default parameter for computing the pseudotime of each cells.

## Results

### An “NK2” subpopulation exhibits the largest distribution divergence between young and elderly individuals

**Fig. 1:**
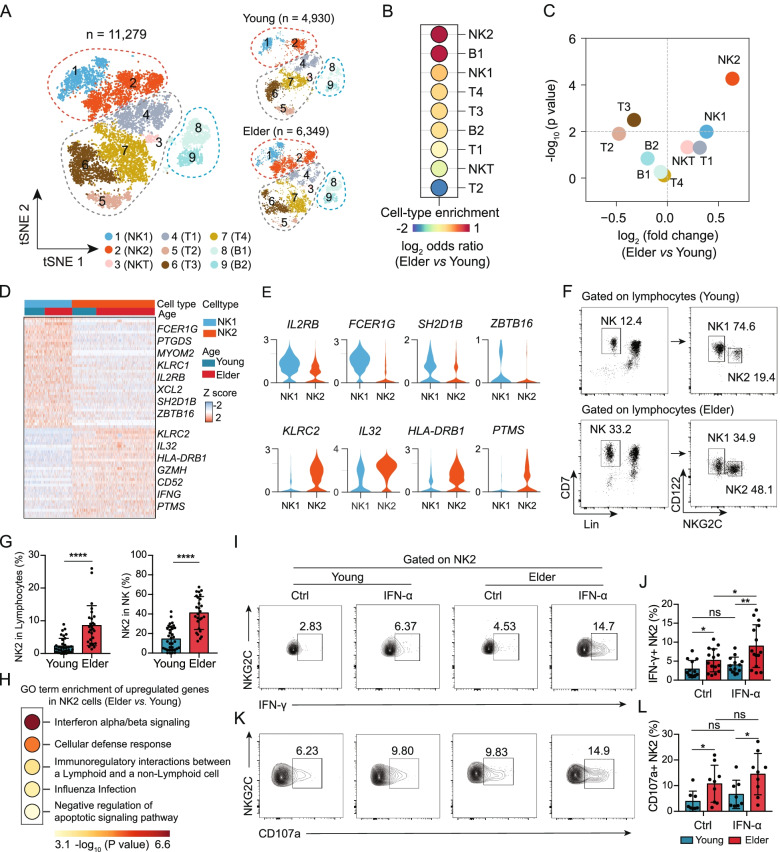
Ageing leads to a marked increase in the proportion of memory-like NK2 cells among total blood lymphocytes. **A** t-SNE representations of the integrated single-cell transcriptomes of 11,279 PBMCs, with 4930 cells from the 4 young individuals (ages 21–28 years) and 6349 cells from the 4 elderly individuals (ages 65–68 years). Cells are coloured by cell type identity. Each dot represents a single cell. **B** Dot plot showing the log_2_ (odds ratio) of the comparison between the cell proportions of each cell cluster in the young and elderly samples. **C** Scatterplot showing the logarithmic ratio between the estimated frequencies of each of the 9 cell clusters in young individuals (*n* = 10) and those in elderly individuals (*n* = 10) from bulk RNA-seq datasets (GEO103232). **D** Heatmap showing the top 30 differentially expressed genes for NK1 and NK2 cells from young and elderly individuals. **E** Violin plot showing the gene expression in NK1 and NK2 cells. **F** FACS staining strategy for NK1 (Lin^-^CD7^+^ NKG2C^−^CD122^high^) and NK2 cells (Lin^−^CD7^+^ NKG2C^+^CD122^low^) from young (top) and elderly (bottom) individuals. **G** Bar graphs showing the proportions of NK2 cells in lymphocytes (left) and NK2 cells in NK cells (right) from young (*n* = 35) and elderly (*n* = 27) individuals. **H** Gene Ontology (GO) enrichment analysis of the differentially expressed genes of NK2 cells from young individuals and those from elderly individuals. The colour indicates the -log_10_ (*P*-value) enrichment for each GO term. **I** Flow cytometry analysis of IFN-γ in NK2 cells from young and elderly individuals with or without IFN-α stimulation in vitro. **J** Bar graphs displaying the frequencies of IFN-γ^+^ in the NK2 subpopulation from young (*n* = 13) and elderly (*n* = 15) individuals with or without IFN-α stimulation in vitro. **K** Flow cytometry analysis of CD107a in NK2 cells from young and elderly individuals with or without IFN-α stimulation in vitro. **L** Bar graphs displaying the frequencies of CD107a^+^ in the NK2 subpopulation from young (*n* = 8) and elderly (*n* = 9) individuals with or without IFN-α stimulation in vitro. The error bars represent the standard deviation (SD). *P < 0.05, **P < 0.01, ****P < 0.0001. *P*-values were obtained with two-sided Student’s *t*-tests. The results are representative of at least three independent experiment

We initially profiled fresh human peripheral blood samples collected from 4 young (ages 21–28 years) individuals and 4 elderly (ages 65–68 years) individuals (Additional file 2: Fig. S1A and Additional file 1: Table S1). Lymphocytes were then sorted and subjected to scRNA-seq using the 10X platform (Additional file 2: Fig. S1B). After rigorous quality control (QC) processing, we retained a total of 11,279 high-quality single transcriptomes (Additional file 2: Fig. S2A, B). Of these, 4930 cells were from young individuals, and 6349 cells were from elderly individuals. We applied Seurat [28] (version 3.2.2) to integrate the single-cell transcriptomes from young and elderly individuals and identified 9 unique immune cell subpopulations, which were visualized via *t*-distributed stochastic neighbour embedding (*t*-SNE) (Fig. 1A). We then applied Souporcell [36], a genotype-based unmixing method that can deconvolve scRNA-seq data for assigning cells to their donor of origin, to help assess the per donor representation of the different cell clusters and to identify any batch effects for individual samples. We obtained 96.6% (10,894/11,279) of cells with individual sample identity and observed that each cell cluster was composed of cells originating from the 4 young and 4 elderly individuals (Additional file 2: Fig. S2C, D), indicating that our resulting clusters were not driven by any single individual.

Based on the expression of known marker genes, we identified lymphocyte lineages, including two NK cell subpopulations (NK1 and NK2), NKT cells, four T cell subpopulations (T1, T2, T3, and T4), and two B cell subpopulations (B1 and B2) (Additional file 2: Fig. S2E and Additional file 1: Table S2). Among the 9 immune cell subpopulations defined in our scRNA-seq data, we found that the proportion of NK2 cells among total lymphocytes was 1.89-fold higher in the elderly than in the young individuals; this was the subpopulation exhibiting the most dramatic enrichment in the distribution in the elderly individuals (Fig. 1B). We also downloaded bulk RNA-seq data for peripheral blood mononuclear cells [32] (PBMCs) from young (*n* = 10) and elderly (*n* = 10) individuals and used the signature genes identified in our single-cell analysis to assess the composition of immune cell subpopulations in this dataset (see the “Methods” section and Additional file 1: Table S3). As in our scRNA-seq data, we again found that the NK2 subpopulation displayed the largest distribution change between the young and elderly individuals (*P* < 10^−4^, two-sided Student’s *t*-tests) (Fig. 1C). These results indicate that the NK2 subpopulation may represent an age-related NK subpopulation in humans.

### NKG2C^+^CD122^low^ NK2 cells increase with ageing and have a memory-like phenotype

We next assessed differentially expressed genes (DEGs) between NK1 and NK2 cells in our scRNA-seq data (Fig. 1D and Additional file 1: Table S4) and noticed that NK2 cells showed reduced expression levels of genes including *FCER1G* (FcεRγ), *SH2D1B* (EAT-2), and *ZBTB16* (PLZF) and elevated expression of *KLRC2* (NKG2C), among others, compared with NK1 cells (Fig. 1E). Since it has been reported that reduced expression of FcɛRγ, PLZF, and EAT-2 and increased expression of NKG2C correlate with a memory NK cell phenotype [37, 38], we considered the NK2 subpopulation to be phenotypically memory-like NK cells.

We further found two surface marker genes, *IL2RB* (also known as *CD122*) and *KLRC2* (*NKG2C*), which can be used to distinguish the NK1 and NK2 subpopulations (Fig. 1E). Using flow cytometry, we confirmed the existence of these two NK cell subpopulations within the Lin^-^CD7^+^ NK cell population in the human blood and characterized NK1 cells as NKG2C^-^CD122^high^ and NK2 cells as NKG2C^+^CD122^low^ (Fig. 1F). Both the two NK cell populations showed high expression (> 95%) of NK-defining surface molecules, including CD56 (Additional file 2: Fig. S3A-D), CD16 (Additional file 2: Fig. S3E-H), and NKp80 (Additional file 2: Fig. S3I-L). We then confirmed in peripheral blood samples from 35 young and 27 elderly healthy donors that the percentages of NK2 cells (NKG2C^+^CD122^low^) among total lymphocytes and among NK cells were significantly higher in the elderly than in the young individuals (Fig. 1G); the percentages of NK1 cells (NKG2C^−^CD122^high^) showed the opposite trend (Additional file 2: Fig. S4A, B).

The expansion of adaptive or memory-like NK cell subsets has been observed in association with the CMV serostatus [39–43]. Therefore, we compared the proportion of NKG2C^+^CD122^low^ memory-like NK2 cells among total blood lymphocytes and NK cells of CMV-seronegative (CMV−) and CMV-seropositive (CMV+) donors (Additional file 2: Fig. S5A-C). Indeed, NKG2C^+^CD122^low^ memory-like NK2 cells were detected more reliably in the blood of CMV+ adult donors (~ 19% in NK cells) than that in CMV− adult donors (~ 5% in NK cells), despite the non-significant differences in the subset distribution of NK cells in the young individuals (Additional file 2: Fig. S5A). It is worth mentioning that the presence of NKG2C^+^CD122^low^ memory-like NK2 cells was correlated with CMV seropositivity, but not all CMV-seropositive donors had detectable (at least by flow cytometry analysis) memory-like NK2 cell subpopulations (Additional file 2: Fig. S5B, C). These results were consistent with those of previous studies showing that not all CMV+ individuals have circulating memory NK cells [37, 44].

Intriguingly, we further confirmed in peripheral blood samples from 35 young and 27 elderly healthy donors that the percentages of NKG2C^+^CD122^low^ memory-like NK2 cells among total lymphocytes and among NK cells were significantly higher in the CMV+ elderly (~ 40% in NK cells) than in the CMV+ young individuals (~ 19% in NK cells) (Additional file 2: Fig. S5B, C). These results suggest a memory-like NK subpopulation exhibiting an age-related increase (considering CMV serostatus), as measured by scRNA-seq and supported by independent flow cytometry results.

Gene Ontology (GO) analysis indicated that compared to NK1 cells, NK2 cells showed enrichment for genes related to lymphocyte activation in our scRNA-seq dataset (Additional file 2: Fig. S6A). We further found that the DEGs of NK2 cells from elderly individuals, compared to young individuals, were enriched for functional annotations related to interferon alpha/beta signalling (Fig. 1H and Additional file 1: Table S5). NK cell effector functions are mediated by CD107a expression and IFN-γ production [45]. We subsequently examined peripheral blood samples from an independent cohort of young and elderly individuals using flow cytometry to detect the secretion of IFN-γ and CD107a in NK1 or NK2 cells during in vitro IFN-α stimulation. Although there were no significant differences in the secretion of IFN-γ and CD107a by NK1 cells when comparing elderly individuals with young individuals upon IFN-α stimulation (Additional file 2: Fig. S6B-E), we found significantly higher IFN-γ and CD107a levels in NK2 cells from elderly individuals than in those from young individuals following in vitro stimulation with IFN-α (Fig. 1I–L and Additional file 1: Table S1).

We also examined the peripheral blood samples from young and elderly individuals using flow cytometry to detect the production of IFN-γ and CD107a in NK1 or NK2 cells upon co-stimulation with interleukin (IL)-12 and IL-15. In young individuals, we found that memory-like NK2 cells displayed decreased responsiveness to IL-12+IL-15 stimulation compared to non-memory-like NK1 cells, as shown by the fact that IL-12+IL-15 induced significantly higher both IFN-γ (Additional file 2: Fig. S7A-C) and CD107a (Additional file 2: Fig. S7D-F) production by NK1 cells than that induced by NK2 cells from young individuals, which is in line with the findings of a previous report [37]. However, in elderly individuals, the responsiveness to IL-12+IL-15 co-stimulation appeared to be similar between memory-like NK2 cells and non-memory-like NK1 cells, because there were no significant differences in the production of IFN-γ and CD107a between NK2 and NK1 cells from elderly individuals (Additional file 2: Fig. S7A-F).

Next, we assessed the functional differences in NK1 and NK2 cells after interaction with classical NK cell targets (K562 cell line). We found that there were no significant differences in the production of IFN-γ and CD107a in NK1 cells upon K562 stimulation between young and elderly individuals (Additional file 2: Fig. S8A-D). Although slightly but significantly higher IFN-γ levels in NK2 cells from elderly individuals than in those from young individuals following in vitro stimulation with K562 (Additional file 2: Fig. S8E, F), no significant difference in the production of CD107a was detected in NK2 cells after interaction with K562 cells between young and elderly individuals (Additional file 2: Fig. S8G, H). These results indicated that age-related changes in human NK cell functionality may not be related to target cell-mediated killing function, but instead to proinflammatory cytokine secretion and type I interferon response status.

Previous studies have reported age-related impairment in IL-2 signalling in NK cells from elderly individuals [46, 47]; we therefore examined the effects of IL-2 on the distinct NK cell population. No significant difference in the production of CD107a and IFN-γ was detected upon IL-2 stimulation of NK1 cells from elderly individuals (Additional file 2: Fig. S9A-D). However, the response to IL-2 in NK2 cells from elderly individuals was found to be impaired when IFN-γ was considered (Additional file 2: Fig. S9E, F), whereas CD107a production was not significantly affected (Additional file 2: Fig. S9G, H). These results provided additional support for the conclusion that NKG2C^+^CD122^low^ memory-like NK2 cells are age-related NK cells.

Taken together, our findings are in line with previous studies reporting that exposure of NK cells to a combination of IL-12 and IL-15 results in memory-like cell behaviours in the absence of antigen, characterized by enhanced effector functions and responses when they are restimulated with cytokines [9, 48]. These findings further support the idea that NK2 cells are phenotypically memory-like NK cells.

### ScRNA-seq of human blood NK cells identifies a unique subset of memory-like NK2.1 cells that is enriched in elderly individuals

**Fig. 2:**
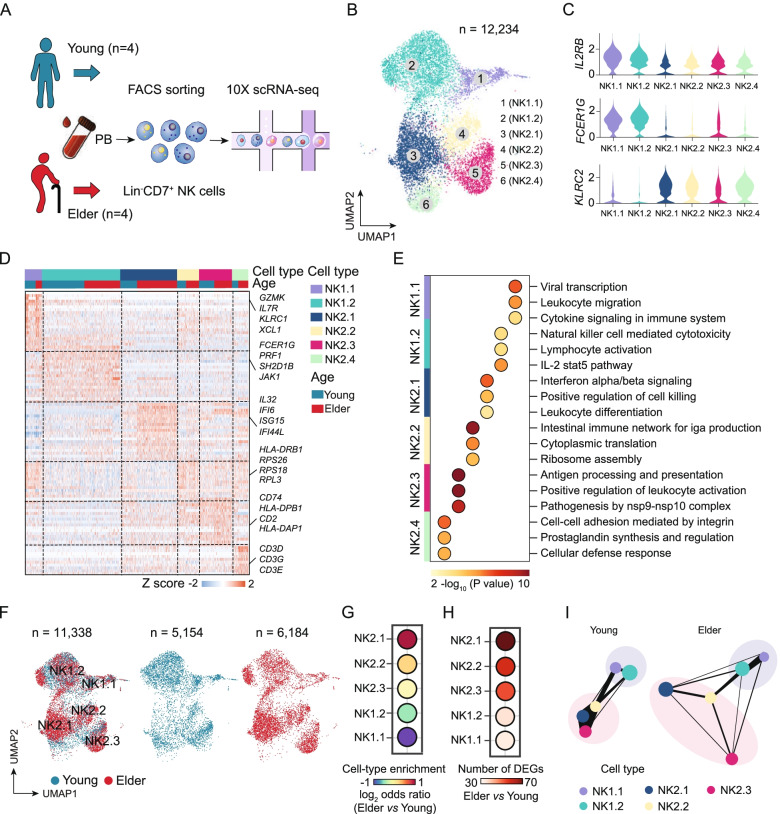
ScRNA-seq analysis of aged human blood NK cells reveals age-associated alterations in memory-like NK cells. **A** Peripheral NK cells (Lin^−^CD7^+^) were sorted from young and elderly individuals and analysed with the 10X Genomics single-cell sequencing platform. **B** UMAP projections of 12,234 NK cells. The different colours represent the 6 NK cell subpopulations (left). Each dot represents a single cell. **C**, Violin plot showing the gene expression of *IL2RB*, *FCER1G*, and *KLRC2* in each cell subpopulation. **D** Heatmap showing the differentially expressed genes among the NK cell subpopulations from young and elderly individuals. **E** GO enrichment analysis showing the terms of the differentially expressed genes for the indicated NK cell subpopulations from young and elderly individuals. The colour indicates the -log_10_ (*P*-value) enrichment for each GO term. **F** UMAP projections of 11,338 NK cells, with 5154 from young individuals (middle) and 6184 cells from elderly individuals (right). Each dot represents a single cell. **G** Dot plot showing the proportions of the NK cell subpopulations in an elder vs. young comparison. **H** Dot plot showing a comparison of the number of differentially expressed genes in the indicated NK cell subpopulations in an elderly vs. young comparison. **I** PAGA graph showing the connectivity between the NK subgroups in the young and elderly groups. Each of the coloured nodes is an NK subpopulation, and the node size is proportional to the number of cells in the subpopulation. The thickness of the edge shows the strength of the connectivity between subpopulations. DEGs, differentially expressed genes

To determine whether additional cellular diversity exists and gain deeper insights into the age-related functional divergence of NK cells, we conducted 10X single-cell transcriptome sequencing on purified NK cells from the same cohort of 4 young and 4 elderly healthy individuals examined above (Fig. 2A). We sorted Lin^−^CD7^+^ NK cells among lymphocytes to obtain cell populations covering all known developmental stages for NK cells and for type 1 innate lymphoid cells (ILCs) [15, 38]. After QC processing, we obtained a total of 12,234 high-quality NK cells, of which 5501 cells were from young individuals and 6733 were from elderly individuals (Additional file 2: Fig. S10A, B). We used Seurat [28] (version 3.2.2) to integrate the young and elderly samples and identified 6 NK cell subsets (namely, NK1.1, NK1.2, NK2.1, NK2.2, NK2.3, and NK2.4 cells), which were represented using uniform manifold approximation and projection (UMAP) (Fig. 2B). We found that compared to both NK1.1 and NK1.2 cells, NK2.1, NK2.2, NK2.3, and NK2.4 cells all exhibited low expression of genes, including *FCER1G* (FcεRγ), *SH2D1B* (EAT-2), and *ZBTB16* (PLZF), and high expression of *KLRC2* (Fig. 2C and Additional file 2: Fig. S10C). This expression pattern correlates with a memory NK cell phenotype*,* suggesting that NK2.1, NK2.2, NK2.3, and NK2.4 cells are phenotypically memory-like NK cells.

We then characterized the potential functional annotations of these memory-like NK cell subsets. GO analysis of subset-defining DEGs (e.g. NK2.1 vs. the other NK cell subsets) among these memory-like NK cell subsets indicated that the upregulated DEGs of NK2.1 cells exhibited enrichment for interferon alpha/beta signalling, with high expression levels of genes including *IL32*, *IFI6*, *ISG15*, and *IFI44L*. The DEGs of NK2.2 cells were enriched for functional annotations related to ribosome assembly, with high expression of genes including *RPS26*, *RPS18*, and *RPL3*. The DEGs of NK2.3 cells showed enrichment for antigen processing and presentation, and the highly expressed genes included *CD74*, *HLA-DPB1*, and *HLA-DAP1* (Fig. 2D, E and Additional file 1: Table S6). Although we performed scRNA-seq analysis using sorted Lin^−^CD7^+^ NK cells and excluded CD3 expression at the protein level, NK2.4 cells still showed *CD3D* and *CD3G* expression at the mRNA level (Fig. 2D and Additional file 2: Fig. S10D). Therefore, we speculate that this cell subset may correspond to previously described activated NKT cells with low expression of the TCR complex [49].

Among the 6 NK cell subsets defined in this scRNA-seq data, we found that the proportion of NK2.1 cells among total NK cells was 1.95-fold higher in the elderly than in the young individuals; this was the subset exhibiting the most dramatic change in distribution between the two age groups (Fig. 2F, G). We next performed pairwise comparisons of the NK cell subsets from elderly individuals and the corresponding cell subsets from young individuals, which identified a total of 253 DEGs (Fig. 2H). When assessing the number of DEGs for each of the NK cell subsets, there were clearly more DEGs in NK2.1 cells than in the other subsets (NK1.1 cells, NK1.2 cells, NK2.2 cells, and NK2.3 cells) (Fig. 2H), suggesting that NK2.1 cells showed the largest transcriptomic changes among NK cells during ageing.

To more intuitively quantify the connectivity of partitions (the NK1.1, NK1.2, NK2.1, NK2.2, and NK2.3 cell subsets) of the single-cell graph, partition-based graphical abstraction (PAGA) [33] was used to generate a much simpler abstracted graph (PAGA graph) of partitions, in which edge weights represent confidence in the presence of connections. We noticed that the connectivity of neighbourhoods appeared to be reduced among NK2.1, NK2.2, and NK2.3 cells from the elderly individuals compared to those from the young individuals (Fig. 2I). Summarizing the above findings, we determined the NK cell hierarchies in the peripheral blood in the context of ageing and identified a unique subset of memory-like NK2.1 cells that is enriched in elderly individuals.

### NK2.1 cells in elderly individuals represent a terminal stage of human NK cell differentiation

**Fig. 3:**
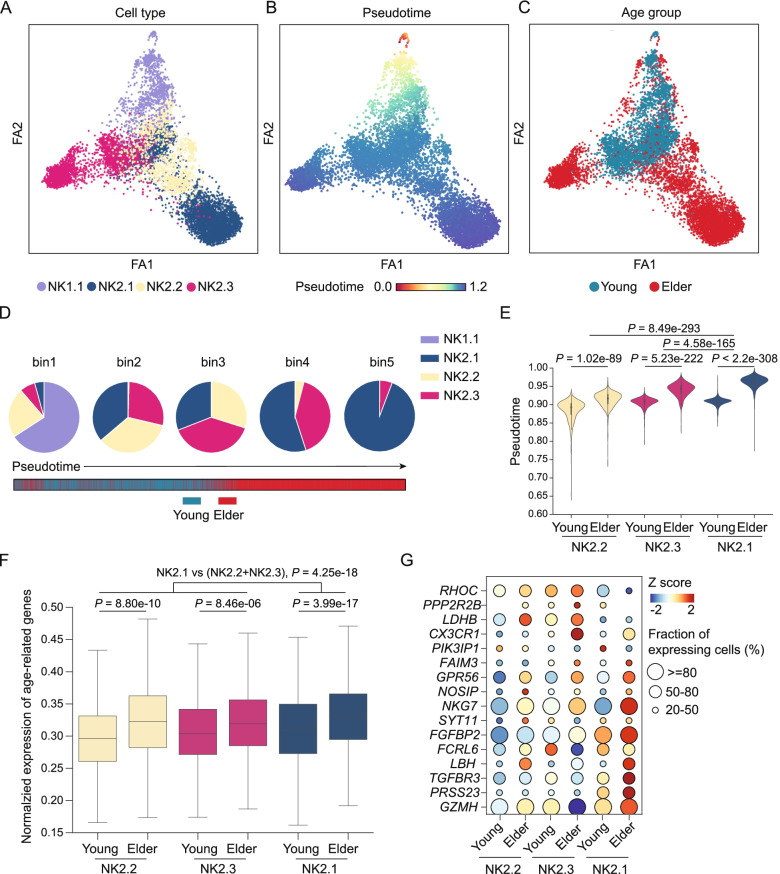
Pseudotime analysis reveals the distinct trajectories of memory-like NK cell differentiation during ageing. **A–C** Trajectories predicted using the PAGA algorithm for NK1.1, NK2.1, NK2.2, and NK2.3 cells from young and elderly individuals. Cells are coloured by NK cell subsets (**A**), by the pseudotime trajectory (**B**), and by age group (**C**). **D** Pie charts showing the composition of NK cell subsets in five bins; each bin was divided equally according to the pseudotime of the cells. **E** Violin plots of the cell pseudotime distribution for each NK cell subset from young and elderly individuals. *P*-values were estimated by Wilcoxon rank-sum test. **F** The box plot shows the expression of the top 50 age-associated genes (obtained from Peters et al. [31]) of NK2.1, NK2.2, and NK2.3 cells from young people and elderly people. *P*-values were obtained using the Wilcoxon rank-sum tests. **G** Dot plot representing the expression of age-related genes of NK2.1, NK2.2, and NK2.3 cells in the young and elderly groups. The plot shows genes expressed by at least 20% of the cells in the subset. The size of the dot represents the fraction of cells expressing the gene, and the colour of the dot represents the gene level as a ***z***-score

The impacts of ageing on the development and maturation of NK cell subsets are not well understood in humans [44]. We applied PAGA [33], a high-resolution pseudotime prediction algorithm, to construct differentiation potential trajectories for NK2.1, NK2.2, and NK2.3 cells from young and elderly individuals. Several experimental evidences have shown that NK cell development proceeds from a CD56^bright^ to CD56^dim^ phenotype [50, 51]. We then tried to define the starting point of the putative developmental trajectory and identified that the NK1.1 cells showed enrichment of gene expression for the CD56^bright^ NK cell signature genes, whereas the NK2.1, NK2.2, and NK2.3 cells were enriched for CD56^dim^ NK cell signature genes (Additional file 1: Table S7 and Additional file 2: Fig. S11A, B). The NK1.1 (CD56^bright^-like NK) subset was, therefore, defined as the starting point of the putative developmental trajectory. Pseudotime analysis with the PAGA algorithm indicated that NK2.1 and NK2.3 cells were distributed on the two branches of the trajectory (Fig. 3A, B). In addition, we found that NK2.1 cells in elderly individuals were projected at the end of one branch along the developmental trajectory (Fig. 3A–C), suggesting that these cells were in the terminal differentiated state in elderly individuals.

To quantitatively illustrate the time order of the three subsets on the pseudotime trajectory, we divided the cells into five equal bins along pseudotime. Notably, ~ 95% of the NK2.1 cells positioned within the terminal pseudotime bin were from elderly individuals (Fig. 3D, E). At minimum, this finding suggests that NK2.1 cells from elderly individuals represent a terminal stage of human NK cell differentiation. A previous study performed a meta-analysis of a large cohort of 14,983 individuals and reported the top 50 age-related genes in human peripheral blood [31] (Additional file 1: Table S8). The constituent genes of this set are related to biological characteristics of personal health (e.g. blood pressure, cholesterol level, fasting blood sugar, and body mass index). Using this gene set for an analysis of each NK2.1, NK2.2, and NK2.3 cell subset, we found that the expression of age-related genes of the NK2.1 cell subset was apparently substantially higher than that of the other two subsets (Fig. 3F, G, *P* = 4.25e−18, Wilcoxon rank-sum test). This suggests that NK2.1 cells represent a terminal differentiation state for NK cells.

### NK2.1 cells in elderly individuals display a transcriptional signature of elevated type I interferon signalling

**Fig. 4:**
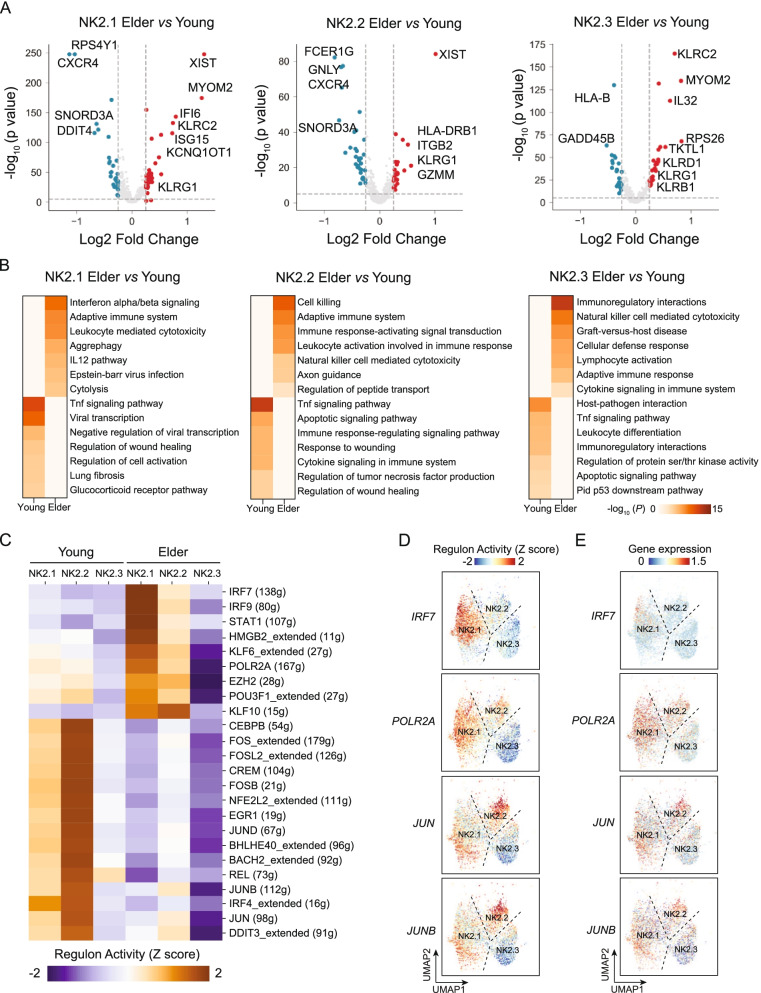
Age-associated transcriptional differences of distinct memory-like NK cell subsets. **A** Volcano plots of the differentially expressed genes of NK2.1 (left), NK2.2 (middle), and NK2.3 cells (right) from comparisons between young and elderly individuals. Each dot represents a gene, with significantly upregulated genes (lnFC > 0.25, *P* < 10^−3^) in young and elderly individuals coloured blue and red, respectively. **B** Heatmap of the enriched GO terms among the DEGs detected for NK2.1, NK2.2, and NK2.3 cells (elderly vs. young). The colour indicates the -log_10_ (*P*-value) enrichment for each GO term. *P*-values were obtained with the Wilcoxon rank-sum tests. **C** Heatmap of the AUC scores predicted by SCENIC for expression regulation by transcription factors (TFs) in NK2.1, NK2.2, and NK2.3 cells from young and elderly individuals. **D**, **E** UMAP plots showing the AUC of the estimated regulon activity for *IRF7*, *POLR2A*, *JUN*, and *JUNB* (**D**) in NK2.1, NK2.2, and NK2.3 cells and the expression of these TFs (**E**). FC, fold change

Transcriptional differences in cells that occur during ageing drive altered functions, so we further investigated the age-related transcriptional differences of the NK2.1, NK2.2, and NK2.3 cell subsets in the DEG analysis between young and elderly samples. We observed that NK2.1 cells from the elderly samples had elevated expression of interferon signalling pathway genes (e.g. *IFI6*, *ISG15*) compared to that of NK2.1 cells from the young samples (Fig. 4A, B and Additional file 1: Tables S9-S10), suggesting that NK2.1 cells may be continuously exposed to interferon signals during ageing.

We also explored transcription factors (TFs) in NK cells that may regulate ageing-associated transcriptional programmes in NK cells. We used SCENIC [34] to predict TFs that may regulate the genes we detected as upregulated in NK2.1, NK2.2, and NK2.3 cells from elderly or young individuals (Fig. 4C and Additional file 2: Fig. S12A). There were 9 SCENIC-predicted TFs—IRF7, IRF9, STAT1, HMGB2, KLF6, POLR2A, EZH2, POU3F1, and KLF10—that appear to affect the observed ageing-associated transcriptomic changes in NK2.1 cells in elderly individuals (Fig. 4C, D and Additional file 2: Fig. S12A, B). IRF7 is a major regulator of type I interferon-dependent immune responses [52]. Notably, the mRNA expression level of *IRF7* was higher in NK2.1 cells from elderly individuals than in NK2.1 cells from young individuals (Fig. 4E). Furthermore, SCENIC-predicted IRF7 may regulate 70.5% (31/44) of the upregulated DEGs in NK2.1 cells in elderly individuals, including the type I interferon signal pathway-related genes *IFI6*, *MX1*, *ISG15*, *IFI44L*, and *IL32* (Additional file 2: Fig. S13A, B). Our finding that IRF7 expression is elevated, in combination with our detection of the enrichment of its motif, in NK2.1 cells from elderly individuals, suggests that this TF may transcriptionally regulate ageing-associated activation of the type I interferon signal transduction pathway.

### NK2.1 cells in elderly individuals are predominantly CD52^+^ NK2 cells, exhibit proinflammatory characteristics, and display a type I interferon response state

**Fig. 5:**
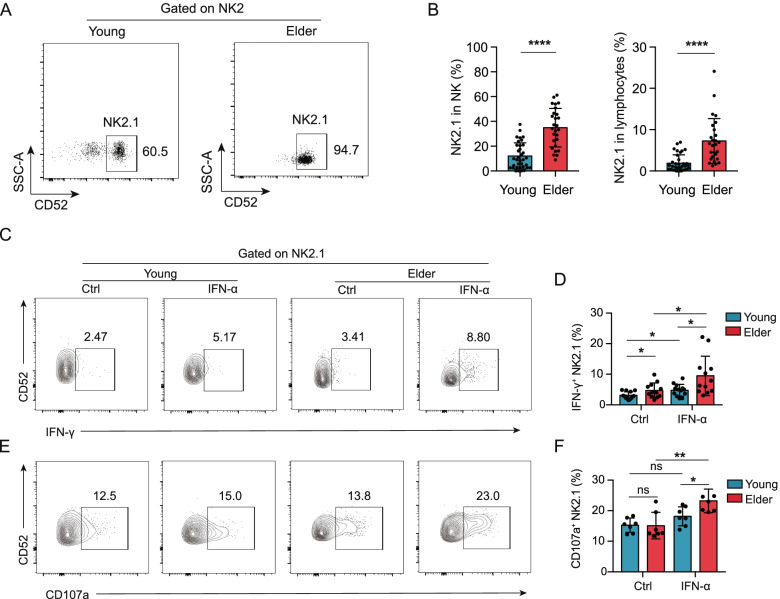
Age-associated memory-like CD52^+^ NK2 cells (NK2.1) exhibit proinflammatory characteristics and display a type I IFN response state. **A** Representative flow cytometry analysis of the percentage of NK2.1 cells within NK2 cells (Lin^−^CD7^+^NKG2C^+^CD122^low^) from young (left) and elderly (right) individuals. **B** Bar graphs showing the proportion of NK2.1 cells among NK cells (left) and among lymphocytes (right) from young (*n* = 35) and elderly (*n* = 27) individuals. **C** Representative density plots showing the expression of IFN-γ in NK2.1 cells from young and elderly individuals with or without IFN-α stimulation in vitro. **D** Bar graphs displaying the frequencies of IFN-γ^+^ cells in NK2.1 cells from young (*n* = 13) and elderly (*n* = 12) individuals with or without IFN-α stimulation in vitro. **E** Representative density plots showing the expression of CD107a in NK2.1 cells from young and elderly individuals with or without IFN-α stimulation in vitro. **F** Bar graphs displaying the frequencies of CD107a^+^ cells in NK2.1 cells from young (*n* = 7) and elderly (*n* = 7) individuals with or without IFN-α stimulation in vitro

Our single-cell transcriptome analysis revealed that NK2.1 cells accumulated significantly in elderly individuals (Fig. 2G). We performed pairwise comparisons among NK2.1, NK2.2, and NK2.3 cells and found that NK2.1 cells had relatively high expression of the *CD52* gene (Additional file 2: Fig. S14A, B). CD52 has been reported as an active target for the management of CMV reactivation [53]. To validate this as a surface marker for NK2.1 cells, we analysed blood samples from 35 young and 27 elderly healthy individuals by flow cytometry with gating for CD52 and for NKG2C and CD122, memory-like NK2 markers identified in our scRNA-seq analysis (Additional file 2: Fig. S15). Beyond supporting the presence of NK2.1 cells (Fig. 5A), this analysis also confirmed that the proportion of NK2.1 (CD52^+^ NK2) cells among NK cells or among total lymphocytes was significantly elevated in elderly individuals (Fig. 5B, *P* < 0.0001, Student’s *t*-tests). NK2.1 cells also showed high expression (> 95%) of NK-defining surface molecules, including CD56 (Additional file 2: Fig. S16A, B), CD16 (Additional file 2: Fig. S16C, D), and NKp80 (Additional file 2: Fig. S16E, F). In addition, when taking CMV serostatus into account, we also found that the percentages of CD52^+^NKG2C^+^CD122^low^ memory-like NK2.1 cells among total lymphocytes and among NK cells were significantly higher in the CMV+ elderly (~ 35% in NK cells) than in the CMV+ young individuals (~ 12% in NK cells) (Additional file 2: Fig. S17A, B).

As NK2.1 cells from elderly individuals exhibited enrichment of genes related to interferon alpha/beta signalling in our scRNA-seq analysis (Fig. 4B), we investigated the response sensitivities of NK2.1 cells from elderly and young individuals to type I interferon stimulation. We analysed the expression levels of IFN-γ and CD107a in NK2.1 cells from an independent cohort of young and elderly individuals following in vitro stimulation with recombinant human IFN-α (Additional file 1: Table S1). NK2.1 cells from the elderly group had significantly higher IFN-γ and CD107a levels than NK2.1 cells from the young group (Fig. 5C–F). Furthermore, NK2.1 cells from the elderly individuals appeared to be more responsive to type I interferon stimulation than young NK2.1 cells, as evidenced by significantly increased levels of IFN-γ and CD107a (Fig. 5C–F). In sum, these results indicate that a marked increase in the proportion of NK2.1 cells—which exhibit proinflammatory characteristics and display a type I interferon response state—might represent an immune cell distribution signature for immune ageing.

### NK2.1 cells from elderly COVID-19 patients are enriched in type I signalling, which is positively correlated with disease severity in COVID-19

**Fig. 6:**
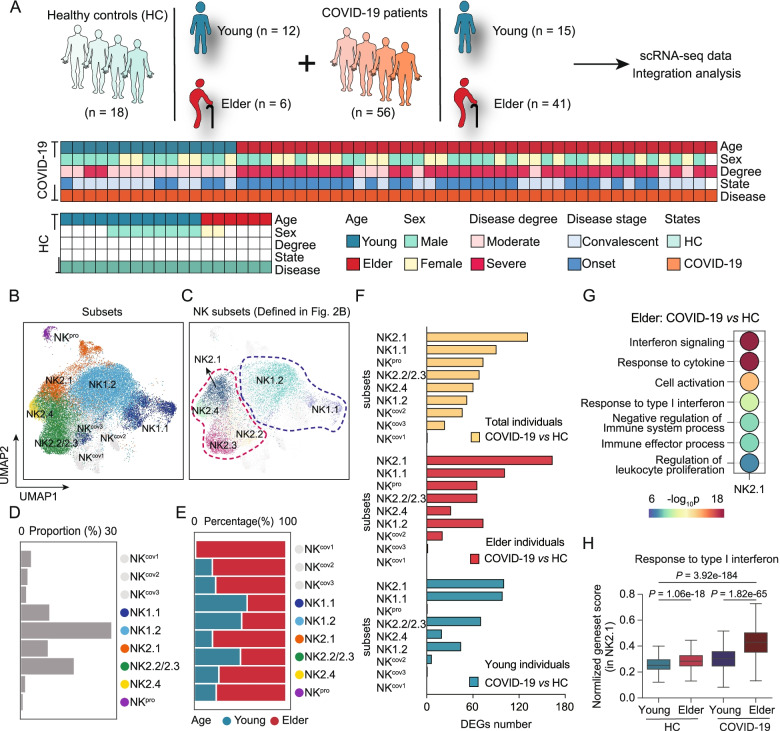
Integrative analyses of a large-cohort COVID-19 single-cell transcriptomic dataset with our single-cell datasets reveal that NK2.1 cells from elderly COVID-19 patients are enriched for type I interferon signalling which correlates with increased disease severity in COVID-19. **A** Flowchart showing the integrative analysis of the scRNA-seq datasets of peripheral blood NK cells obtained from healthy controls (HC, *n* = 18) and COVID-19 patients (*n* = 56) [23, 25, 56]. **B**, **C** UMAP projections of the integrated single-cell transcriptomes of 34,388 NK cells, with 17,748 cells from healthy control individuals and 16,640 cells from COVID-19 patients. Cells are coloured by subset identity (**B**), and the NK cell subsets were defined as in Fig. 2B (**C**). Each dot represents a single cell. **D** Bar graph showing the proportion of each NK cell subset in all cells. **E** Bar graph showing the proportion of cells derived from young or elderly samples for each of the NK cell subsets. **F** Histograms showing the number of DEGs for each NK cell subset in the total individuals (top), the elderly individuals (middle), and the young individuals (bottom) between COVID-19 patients and healthy controls. **G** Dot plots of enriched GO terms of differentially expressed genes among NK2.1 cells between elderly COVID-19 patients and elderly healthy control individuals. The colour of the dot indicates -log_10_ (*P*-value) enrichment for each GO term, and the size of the dot indicates the number of differentially expressed genes contained within each enriched GO term. **H** Box plots of the average expression of genes involved in the signalling pathway “response to type I interferon” in NK2.1 cells from young healthy control individuals, from elderly healthy control individuals, from young COVID-19 patients, and from elderly COVID-19 patients

Studies have shown that the risk for severe COVID-19 illness increases with age [54] and that NK cells undergo enhanced effector functional changes in COVID-19 patients [55]. However, the impacts of ageing on NK cell subsets in COVID-19 disease remain unclear. We downloaded single-cell RNA-seq datasets [23–25, 56] of peripheral immune cells from young (*n* = 15) and elderly (*n* = 41) COVID-19 patients and from young (*n* = 12) and elderly (*n* = 6) healthy control individuals. Specifically, the COVID-19 single-cell datasets included 41 samples taken from elderly patients with active disease (from severe disease, *n* = 25; from moderate disease, *n* = 1) and during the convalescent phase (from severe disease, *n* = 9; from moderate disease, *n* = 6) and 15 samples taken from young patients with active disease (with moderate disease, *n* = 5) and during the convalescent phase (from severe disease, *n* = 2; from moderate disease, *n* = 8) (Fig. 6A and Additional file 1: Table S11).

We then extracted the single-cell transcriptomes of NK cells from COVID-19 patients and healthy control individuals and applied Seurat [28] (version 3.2.2) to integrate the COVID-19 single-cell transcriptomes of NK cells with those from healthy control individuals, enabling the analysis of a total of 34,388 NK cells (19,833 cells from elderly individuals and 14,555 from young individuals). We identified 9 NK cell subsets, which were represented using UMAP (Fig. 6B); note that by mapping our aforementioned NK cell subsets (Fig. 2B) to this UMAP, we found that 7 of the 9 subsets were coincident with NK cell subsets in the aforementioned single-cell analysis, and 2 of the 9 subsets appeared to be COVID-19-specific NK cells (Fig. 6B, C), albeit with low cell numbers (Fig. 6D). We again found that the proportion of NK2.1 among total NK cells was higher in the elderly than in the young COVID-19 patients and higher in the elderly than in the young healthy control individuals (Fig. 6E). Previous studies showed that the proportion of memory NK cells from COVID-19 patients is elevated in severe disease compared to moderate disease [55, 57]. Our integrative scRNA-seq data demonstrated that the percentage of memory-like NK2 cells among total NK cells was increased in elderly patients with severe disease compared to elderly patients with moderate disease (Additional file 2: Fig. S18).

Differential analysis of each NK cell subset between COVID-19 patients and healthy controls showed that there were few DEGs (*n* = 75) for NK cell subsets from young samples but many DEGs (*n* = 357) for NK cell subsets from elderly samples. Furthermore, we identified NK2.1 cells that showed the largest number of DEGs among all NK cell clusters in a comparison of elderly COVID-19 patients and elderly healthy controls (Fig. 6F and Additional file 2: Fig. S19), suggesting disease progression-related functions in NK2.1 cells from elderly COVID-19 patients. We also compared the predicted functions of NK2.1 cells in COVID-19 patients compared with healthy controls, and GO analysis indicated that the DEGs of NK2.1 cells from elderly COVID-19 patients, compared to elderly healthy controls, were enriched for functional annotations related to response to type I interferon (Fig. 6G, H and Additional file 1: Table S12; Additional file 2: Fig. S20A and Additional file 1: Table S13, *P* = 8.20e−35, Wilcoxon rank-sum test). We further found that age has a strong influence on the expression of genes related to type I interferon responses in COVID-19 patients (Additional file 2: Fig. S20B). Specifically, the NK2.1 cells of elderly patients have high expression of genes with functional annotations related to response to type I interferon (e.g. *ISG15*, *ISG20*, etc.) compared to that of NK2.1 cells from young COVID-19 patients (Fig. 6H, *P* = 1.11e−36, Wilcoxon rank-sum test).

Because type I interferon molecules are known to exhibit a wide range of antiviral activities and given the numerous reports of more severe type I interferon responses in patients with severe COVID-19 [58–60], multiple clinical trials have used such molecules as potential therapeutic agents to treat COVID-19. We observed elevated expression of genes involved in response to type I interferon in NK2.1 cells from elderly COVID-19 patients in the severe stage compared with those from elderly COVID-19 patients in the moderate stage (Additional file 2: Fig. S20B, C). In contrast, the expression of genes involved in response to type I interferon in NK2.1 cells from young COVID-19 patients in the severe stage was significantly lower than that in NK2.1 cells from young COVID-19 patients in the moderate stage (Additional file 2: Fig. S20B, C). These contrasting trends suggest that the effect of type I interferon signalling on NK2.1 cells might differ for COVID-19 patients in an age-related manner. Together, these results indicate that NK2.1 cells of elderly COVID-19 patients showed enrichment of type I interferon responses which was positively correlated with the disease severity.

## Discussion

In the present study, we assessed age-related changes in human NK cell subpopulations at single-cell resolution. We uncovered a subpopulation of memory-like NK2 cells that exhibited the largest distribution change between elderly and young individuals among 9 blood immune cell subpopulations. In particular, we discovered a unique NK subset of NK2.1 cells, which were predominantly CD52^+^ NK2 cells, accumulated with age and exhibited proinflammatory characteristics. In addition, NK2.1 cells in elderly individuals displayed elevated sensitivity to type I interferon stimulation in vitro. The potential impact of this cell population on infectious diseases in elderly individuals was evident in our observation that NK2.1 cells from elderly COVID-19 patients are enriched in type I signalling, which we found to be positively correlated with disease severity in COVID-19. Several scRNA-seq studies have been performed in human NK cells, including those from the tonsils [61], bone marrow [38, 62], spleen [63], and blood [38]. However, to the best of our knowledge, unbiased scRNA-seq analyses revealing age-related changes in human NK cells have not been reported. Although efforts have been made to investigate age-related changes in NK cells, such studies were mostly performed using flow cytometry and have been interpreted in the conceptual framework of traditional CD56^dim^ NK cells and CD56^bright^ NK cells.

Our initial scRNA-seq analysis of lymphocytes from young and aged human blood in the present study identified an NK2 subpopulation that exhibits the largest distribution change between elderly and young individuals among 9 blood immune cell subpopulations. This NK2 subpopulation featured high expression of NKG2C KLRC2) and low expression of PLZF (PLZF), FcεRγ (FCER1G), EAT-2 (SH2D1B), and SYK (*SYK*), which are used as markers for memory NK cells in the context of human cytomegalovirus (HCMV) infection [37, 64]. Interestingly, Yang et al. [38] recently reported an “memory-like” NK cell subpopulation in human bone marrow, with memory transcriptional signatures similar to those of the NK2 cells we identified here, although they did not find this NK subpopulation in their human blood dataset.

A previous study has investigated age-related changes in NK cells [65]. Some CD56^dim^CD57^+^ NK cell subsets increased with age, whereas CD56^dim^NKG2C^+^ did not [21, 65–68]. Recently, a longitudinal study demonstrated that the CD57^+^NKG2C^+^CD56^dim^ NK cells may be driven less by chronological ageing, and far more by CMV infection [69]. In the present study, we identified memory-like NKG2C^+^CD122^low^ NK2 cells that increased with ag*e*ing (considering CMV serostatus), as measured by scRNA-seq and supported by independent flow cytometry results. In humans, the expansion of memory-like NK cells has been linked to CMV serostatus [39–42]. So far, it has proved difficult to disentangle the effects of CMV infection and the ageing process that makes CMV infection one of the driving forces of so-called inflammaging. However, it may in fact be primarily associated with CMV serostatus and secondarily with age, linking the increased frequency of CMV infection to age. Whether ageing, along with CMV seropositivity, contributes to a marked increase in the proportion of NKG2C^+^CD122^low^ memory-like NK cells remains to be investigated.

Our analysis of scRNA-seq data collected for purified Lin^−^CD7^+^ NK cells from young and aged human blood further identified a unique subset of memory-like NK2.1 cells. Notably, NK2.1 cells accumulated with age. Intriguingly, these memory-like NK2.1 cells from elderly individuals had higher IFN-γ and CD107a levels than NK2.1 cells from young individuals following in vitro stimulation with IFN-α. This is consistent with the previous observation that persistent memory-like NK populations show high IFN-γ production and potent cytotoxic activity upon ex vivo restimulation [6]. Furthermore, a recent study [70] demonstrated that compared to “younger” NK cells, “older” mouse NK cells exhibited more potent IFN-γ production upon exposure to activating stimuli, as well as more robust adaptive responses during CMV infection. Consistently, we showed here that memory-like NK2.1 cells from elderly individuals appeared to be more responsive to type I interferon stimulation than NK2.1 cells from young individuals, as evidenced by significantly increased levels of cytokines (IFN-γ and CD107a). This implies potential age-related impacts on the functions of memory NK cell-mediated responses. It is well known that a main feature of the ageing process is chronic low-grade inflammation, with elevated levels of proinflammatory cytokines; this is collectively referred to as “immune ageing” [4, 71]. Since memory-like NK2.1 cells accumulate with age and exhibit proinflammatory characteristics, it is plausible that a marked increase in NK2.1 cells can be understood as a signature of immune ageing in humans. Therefore, our study provides important insights into how ageing influences human NK cells, represents a conceptual advance in immune ageing research and helps drive investigations into immune ageing.

It has been shown that type I IFNs positively regulate NK cell activation and cytotoxicity, as well as maturation and memory, and these effects are induced through interferon-stimulating genes (ISGs) [72]. It has also been shown that blocking type I IFN signals can partially restore cognitive deficits caused by ageing [73]. Strikingly, we found that aged memory-like NK2.1 cells exhibited enrichment for genes related to type I IFN signalling, and these cells had 9 high-confidence regulons that were predicted to be governed by TFs, including the IRF7, IRF9, and STAT1, proteins, which were each previously shown to function as key regulators of type I IFN signalling [74]. This is in line with the established dual-phase programme that is coordinately regulated by the TFs IRF and STAT, specifically IRF3/IRF7 for IFN production [74] and STAT1-STAT2-IRF9 for ISG expression [75]. Future studies can dissect the mechanism(s) underlying the apparent age-related accumulation of this unique memory-like NK2.1 subset.

The ongoing COVID-19 pandemic has infected over 100 million people, and there have been more than 2 million deaths to date [76]. COVID-19 patients display high levels of inflammatory cytokines and chemokines [77], especially severe-stage patients, who are known to exhibit elevated expression of TNFα, IL-1, IL-6, IL-18, IL-8, IL-10, MCP-1, and MIP-1α, supporting that idea systemic inflammation and infection is evident in the peripheral blood of COVID-19 patients. Additionally, elderly adults are at higher risk for severe illness from COVID-19, but the underlying immunopathogenic mechanisms remain unclear. Recently, several studies have shed light on the role of NK cells in COVID-19 disease progression [25, 55–57, 78]. A detailed map of NK cell activation in COVID-19 disease revealed that CD56^bright^ NK cell arming is associated with the disease severity in COVID-19 [55, 57]. In addition, a recent study showed that NK cells in early severe COVID-19 display signs of a strong IFN-α response with increased expression of IFN-stimulated genes and genes related to IFN-α signalling [56]. In the present study, considering both age and disease status, we performed an integrative analysis of a large-cohort COVID-19 single-cell transcriptomic dataset with our single-cell datasets [23, 25, 56]. We analysed a total of 34,388 NK cells (19,833 cells from 47 elderly individuals and 14,555 cells from 27 young individuals), which also allowed for cross-validation of our findings. Integrating our findings with earlier reports [23, 25, 56], we further revealed that aged NK2.1 cells are enriched for type I interferon signalling molecules; we found that this effect is positively associated with disease severity in elderly COVID-19 patients. These data are in line with previous observations that patients with more severe COVID-19 disease status exhibit elevated activation of type 1 interferon signalling [56, 59, 79].

The role of type I interferons in the pathogenesis or protection in severe vs. moderate COVID-19 is still somewhat unclear, as some studies have reported impaired type I interferon activity and inflammatory responses in severe COVID-19 patients [80, 81]. Whether the proinflammatory features of NK2.1 cells, particularly type I interferon signalling in NK2.1 cells, can contribute to the pathogenesis of COVID-19 remains to be investigated.

Overall, our study revealed that a marked increase in memory-like NK cells, particularly NK2.1 cells, is a signature of immune ageing in humans. This memory-like NK2.1 subset represents a potential target for the development of immunotherapies to treat infectious diseases and for addressing age-related dysfunctions of the immune system. Our single-cell transcriptomic data can serve as a resource for understanding human NK cell biology during ageing and can help drive investigations into immune ageing.

## Conclusions

We performed a detailed characterization of aged human NK cells using single-cell RNA sequencing, together with functional studies. We identified a unique memory-like NK cell subset that accumulated with ageing and displayed a type I interferon response state. We further performed integrative analyses of a large cohort COVID-19 single-cell transcriptomic dataset with our single-cell datasets and revealed that the type I interferon-responsive memory-like NK cell subset positively correlated with disease severity in COVID-19. These data provide important insights into how ageing influences human NK cells and help drive investigations into immune ageing.

## Supplementary Information

**Additional file 1: Table S1.** Sample information. **Table S2.** Lymphocyte cluster marker genes. **Table S3.** Bulk RNA-seq DEseq normalization data in Fig. 1C. **Table S4.** NK1 vs NK2 DEGs in Fig. 1D. **Table S5.** NK1 vs NK2 GO term in Fig. 1H. **Table S6.** NK cell subsets marker genes in Fig. 2D. **Table S7.** Gene-sets of CD56^bright^ NK cell signature genes and CD56^dim^ NK cell signature genes. **Table S8.** Top50 age-related genes in Fig. 3F. **Table S9.** DEGs in NK2.1, NK2.2, and NK2.3 cells (Elderly vs Young). **Table S10.** GO term in NK2.1, NK2.2, and NK2.3 cells (Elderly vs Young). **Table S11.** COVID-19 patient information in Fig. 6A. **Table S12.** GO term of upregulated genes (Elderly COVID-19 patients vs elderly healthy controls) in Fig. 6G. **Table S13.** Interferon alpha/beta signaling genes.

**Additional file 2: Fig. S1.** Experimental design and flow cytometry sorting strategy. **Fig. S2.** Quality control of single-cell data for lymphocytes from young and elderly individuals. **Fig. S3.** Expression levels of NK-defining surface molecules (*i.e.*, CD56, CD16, and NKp80) in gated NK1 and NK2 cells from young and elderly individuals. **Fig. S4.** The proportional decreases in NK1 cells in elderly individuals. **Fig. S5.** NKG2C^+^CD122^low^ NK2 cells expand in CMV seropositive elderly individuals. **Fig. S6.** NK2 cells in elderly individuals secrete elevated levels of IFN-γ and CD107a. **Fig. S7.** Expression of IFN-γ and CD107a in NK1 cells and NK2 cells from young and elderly individuals. **Fig. S8.** Expression levels of IFN-γ and CD107a in Lin^-^CD7^+^CD122^+^NKG2C^-^ NK1 and in Lin^-^CD7^+^CD122^-^NKG2C^+^ NK2 cells from young and elderly individuals with or without K562 stimulation *in vitro*. **Fig. S9.** Expression levels of IFN-γ and CD107a in Lin^-^CD7^+^CD122^+^NKG2C^-^ NK1 and in Lin^-^CD7^+^CD122^-^NKG2C^+^ NK2 cells from young and elderly individuals with or without IL-2 stimulation *in vitro*. **Fig. S10.** Quality control of single-cell data for NK cells from young and elderly individuals. **Fig. S11.** Identification of NK cell subsets. **Fig. S12.** Age-associated transcription factors of distinct NK cell subsets. **Fig. S13.** Intersection of IRF7 binding genes predicted by SCENIC and upregulated genes in NK2.1 cells from a comparison of elderly vs. young individuals. **Fig. S14.** Differentially expressed genes in NK2.1, NK2.2, and NK2.3 cells. **Fig. S15.** FACS gating strategy for Lin^-^CD7^+^CD122^-^NKG2C^+^ CD52^+^ NK2.1 cells from young and elderly individuals. **Fig. S16.** Expression levels of NK-defining surface molecules (*i.e.*, CD56, CD16, and NKp80) in gated NK2.1 cells from young and elderly individuals. **Fig. S17.** CD52^+^NKG2C^+^CD122^low^ NK2.1 cells expand in CMV seropositive elderly individuals. **Fig. S18.** NK cell subsets in COVID-19 patients. Pie chart showing the proportions of the 9 NK subsets among NK cells from young (top) and elderly (bottom) COVID-19 patients. **Fig. S19.** NK2.1 cells show the largest number of DEGs in a comparison of elderly severe-stage/active-disease COVID-19 patients and elderly healthy controls. **Fig. S20.** Alpha/beta interferon signalling in the NK2.1 cell subset positively correlates with disease progression and age.

## Data Availability

The raw scRNA-seq data for lymphocytes and NK cells from the blood of young and elderly individuals used in this study have been deposited in the Genome Sequence Archive Human (GSA Human) under accession number HRA000632, which is accessible at https://ngdc.cncb.ac.cn/gsa-human/browse/HRA000632 [82]. The processed gene expression data and the raw scRNA-seq data in this study have been deposited into the NCBI GEO database: GSE199337 (https://www.ncbi.nlm.nih.gov/geo/query/acc.cgi?acc=GSE199337) [83]. The code used for data analysis in the article and the processed matrix are available at the following GitHub repository: https://github.com/QuKunLab/Aging-NK [84]. The scRNA-seq data of COVID-19 were obtained from the Gene Expression Omnibus (GEO) database under accession numbers GSE158055 (https://www.ncbi.nlm.nih.gov/geo/query/acc.cgi?acc=GSE158055) [23], GSE184329 (https://www.ncbi.nlm.nih.gov/geo/query/acc.cgi?acc=GSE184329) [25], and from European Genome-phenome Archive (EGA) under accession number EGAS00001004571 (https://ega-archive.org/datasets/EGAD00001006550/files) [24]. The bulk RNA-seq datasets of PBMCs were downloaded from GSE103232: https://www.ncbi.nlm.nih.gov/geo/query/acc.cgi?acc=GSE103232 [32]. The code related to the analyses is available from GitHub (SingleR: https://github.com/dviraran/SingleR [26], Seurat: https://github.com/satijalab/seurat [28], and SCENIC: https://github.com/aertslab/SCENIC [34]
