# Supplementary material for: Single-cell transcriptomics reveal a unique memory-like NK cell subset that accumulates with ageing and correlates with disease severity in COVID-19
Source: Genome Med. 2022 May 3;14:46. doi: 10.1186/s13073-022-01049-3 (PMC9060844; doi:10.1186/s13073-022-01049-3)
Supplement: Supplementary file 2 — Additional file 2: Fig. S1. Experimental design and flow cytometry sorting strategy. Fig. S2. Quality control of single-cell data for lymphocytes from young and elderly individuals. Fig. S3. Expression levels of NK-defining surface molecules (i.e., CD56, CD16, and NKp80) in gated NK1 and NK2 cells from young and elderly individuals. Fig. S4. The proportional decreases in NK1 cells in elderly individuals. Fig. S5. NKG2C+CD122low NK2 cells expand in CMV seropositive elderly individuals. Fig. S6. NK2 cells in elderly individuals secrete elevated levels of IFN-γ and CD107a. Fig. S7. Expression of IFN-γ and CD107a in NK1 cells and NK2 cells from young and elderly individuals. Fig. S8. Expression levels of IFN-γ and CD107a in Lin-CD7+CD122+NKG2C- NK1 and in Lin-CD7+CD122-NKG2C+ NK2 cells from young and elderly individuals with or without K562 stimulation in vitro. Fig. S9. Expression levels of IFN-γ and CD107a in Lin-CD7+CD122+NKG2C- NK1 and in Lin-CD7+CD122-NKG2C+ NK2 cells from young and elderly individuals with or without IL-2 stimulation in vitro. Fig. S10. Quality control of single-cell data for NK cells from young and elderly individuals. Fig. S11. Identification of NK cell subsets. Fig. S12. Age-associated transcription factors of distinct NK cell subsets. Fig. S13. Intersection of IRF7 binding genes predicted by SCENIC and upregulated genes in NK2.1 cells from a comparison of elderly vs. young individuals. Fig. S14. Differentially expressed genes in NK2.1, NK2.2, and NK2.3 cells. Fig. S15. FACS gating strategy for Lin-CD7+CD122-NKG2C+ CD52+ NK2.1 cells from young and elderly individuals. Fig. S16. Expression levels of NK-defining surface molecules (i.e., CD56, CD16, and NKp80) in gated NK2.1 cells from young and elderly individuals. Fig. S17. CD52+NKG2C+CD122low NK2.1 cells expand in CMV seropositive elderly individuals. Fig. S18. NK cell subsets in COVID-19 patients. Pie chart showing the proportions of the 9 NK subsets among NK cells from young (top) and elderly [file 13073_2022_1049_MOESM2_ESM.doc]

**Figure S1**

**
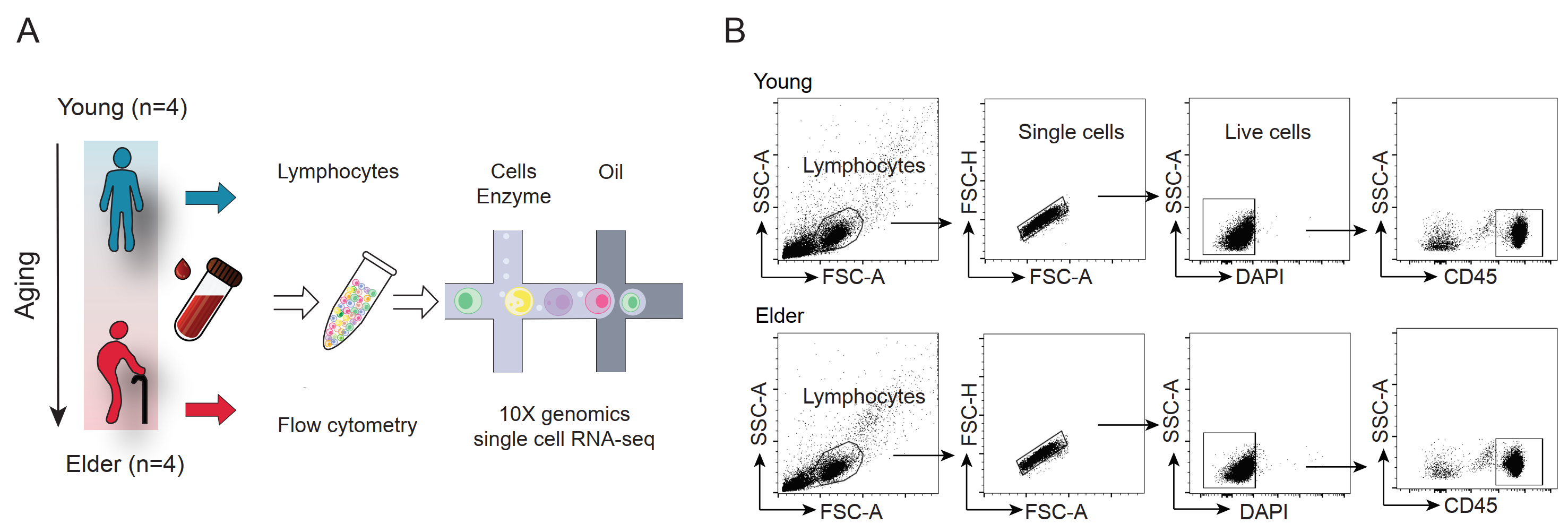
**

**Fig. S1 | Experimental design and flow cytometry sorting strategy.**

**A,** Schematic of the experimental design. Lymphocytes were sorted from the peripheral blood of young or elderly individuals and then analysed with the 10X Genomics single-cell sequencing platform. **B,** FACS staining strategy for CD45^+^DAPI^-^ cells in samples from young individuals (top) and elderly individuals (bottom).

**Figure S2**


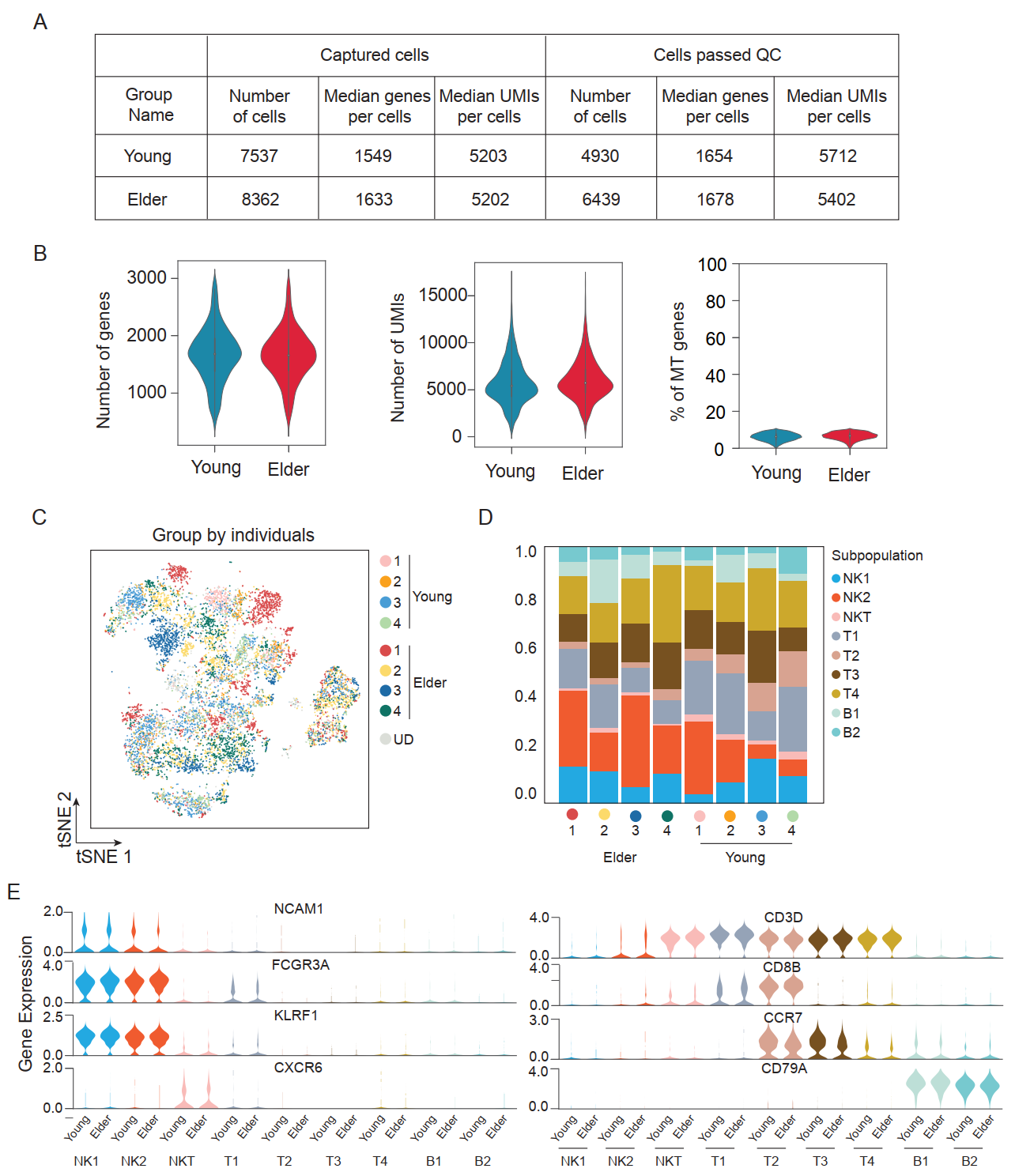


**Fig. S2 | Quality control of single-cell data for lymphocytes from young and elderly individuals. A,** Summary of captured cells, median genes per cell, median UMIs per cell, and the number of cells that passed quality control (QC) criteria for the single-cell data**. B,** Violin plots showing the gene number, UMI number, and percentage of mitochondrial RNA in single-cell data from young and elderly individuals. **C**, t-SNE representations of the integrated single-cell transcriptomes of 11,279 PBMCs. Cells are colored by their donor of origin predicted by Souporcell (Heaton, H. et al. *Nature Methods*, 2020). Each dot represents a single cell. UD, undefined cells. **D**, The stacked bar chart showing the proportion of NK1, NK2, NKT, T1, T2, T3, T4, B1, and B2 cells for each young and elderly individual. **E,** Violin plots showing the expression levels of selected marker genes from the cell clusters.

**Figure S3**

**
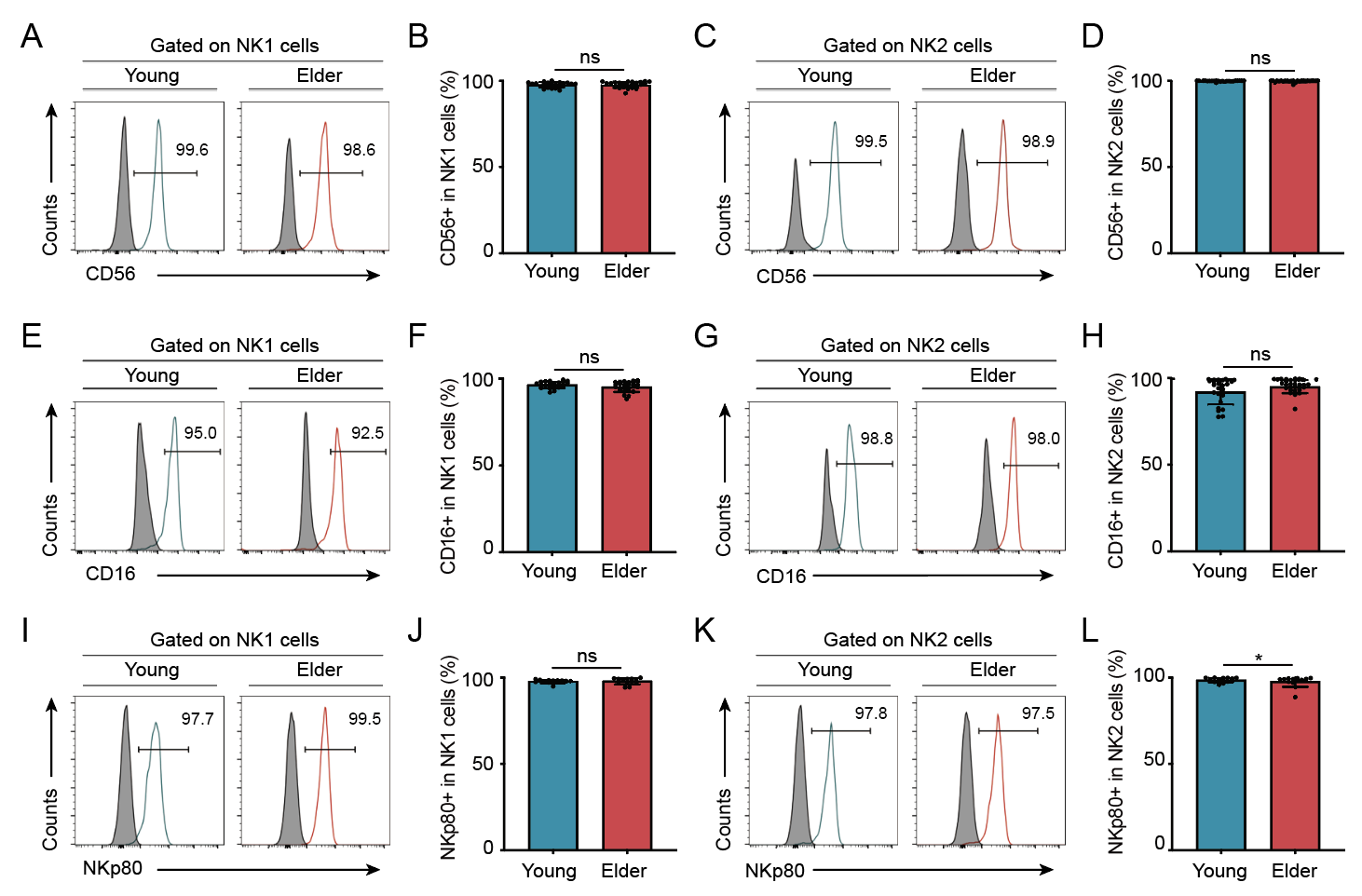
**

**Fig. S3 | Expression levels of NK-defining surface molecules (*i.e.*, CD56, CD16, and NKp80) in gated NK1 and NK2 cells from young and elderly individuals.** **A**, **E**, **I**, FACS staining strategy for CD56 (A), CD16 (E), and NKp80 (I) expression in gated NK1 cells from young and elderly individuals. **B**, **F**, **J**, Bar plots showing the percentage of CD56 (B), CD16 (F), and NKp80 (J) expression in gated NK1 cells from young (n=14-23) and elderly individuals (n=13-25). **C**, **G**, **K**, FACS staining strategy for CD56 (C), CD16 (G), and NKp80 (K) expression in gated NK2 cells from young and elderly individuals. **D**, **H**, **L**, Bar plots showing the percentage of CD56 (D), CD16 (H), and NKp80 (L) expression in gated NK2 cells from young (n=14-23) and elderly individuals (n=13-25). * *P* < 0.05; ns, not significant. Student’s t-test.

**Figure S4**


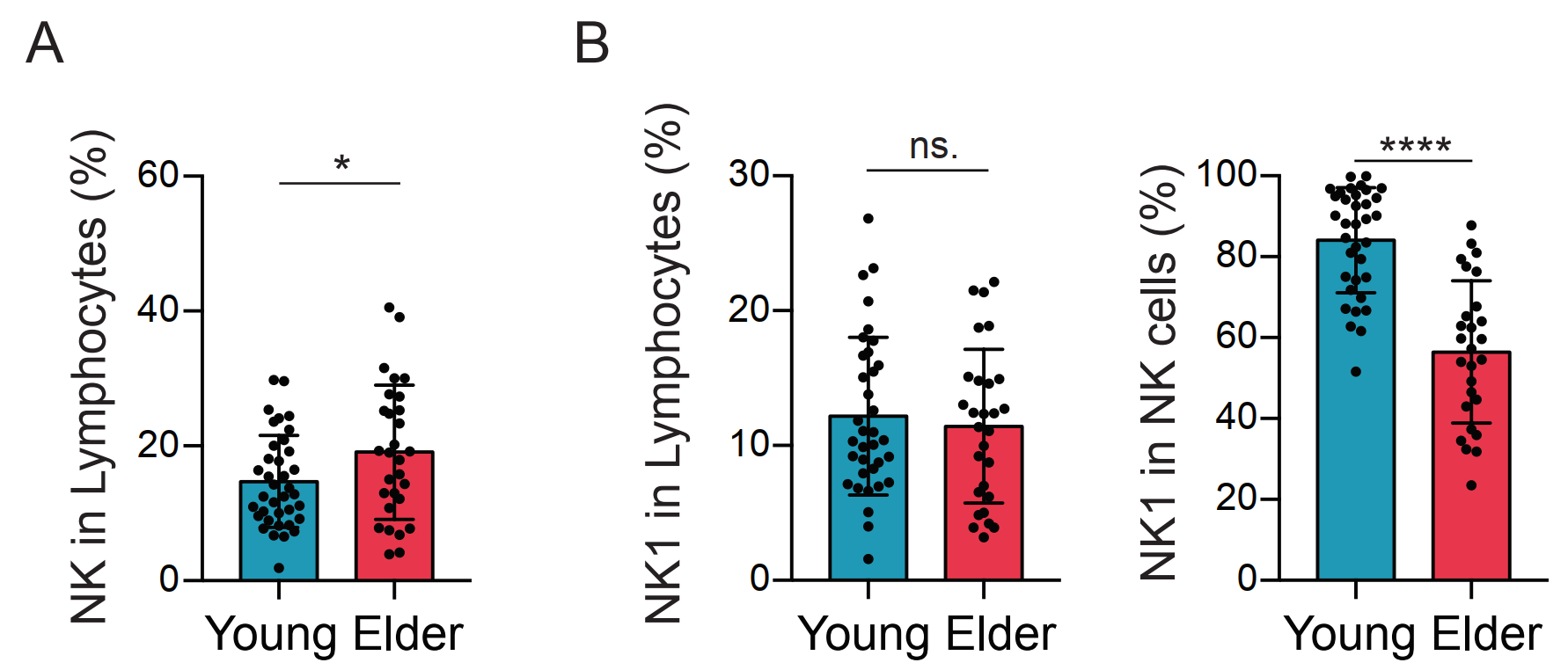


**Fig. S4 | The proportional decreases in NK1 cells in elderly individuals. A,** Histogram showing the proportion of Lin^-^CD7^+^ NK cells in lymphocytes from young and elderly individuals. **B,** Histogram showing the proportion of NK1 in lymphocytes (left) and in NK cells (right) from young (n=35) and elderly individuals (n=27). Error bars represent the standard deviation (SD). * *P* < 0.05, **** *P* < 0.0001. ns. not significant. *P*-values were obtained from two-sided Student’s *t*-tests.

**Figure S5**


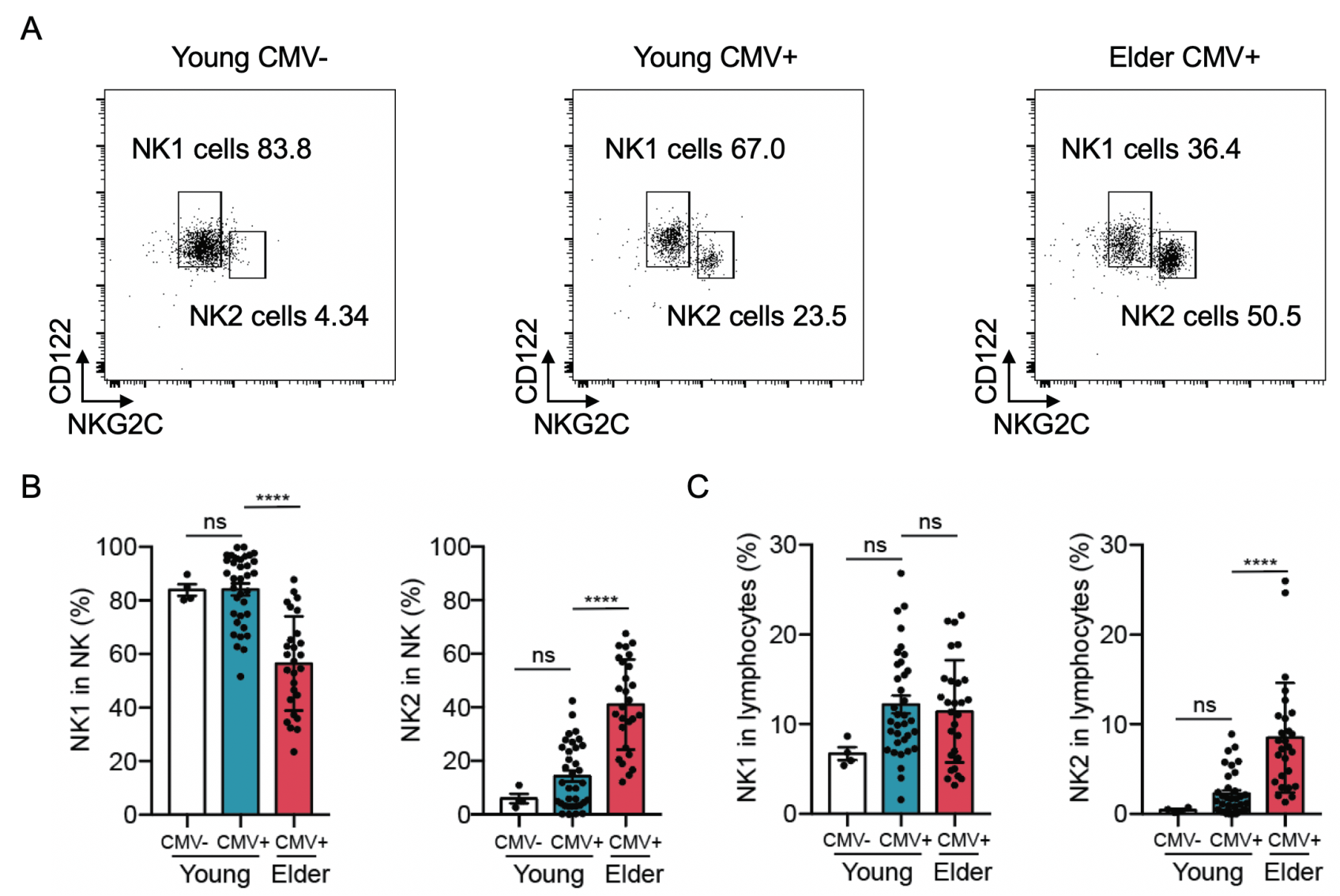


**Fig. S5 | NKG2C^+^CD122^low^ NK2 cells expand in CMV seropositive elderly individuals.** **A**, FACS staining strategy for NK1 (Lin-CD7^+^NKG2C^-^CD122^high^) and NK2 cells (Lin^-^CD7^+^ NKG2C^+^CD122^low^) from CMV seronegative young (Young CMV-), CMV seropositive young (Young CMV+), and CMV seropositive elderly (Elder CMV+) individuals. **B, C,** Bar plots showing the proportions of NK1 cells and NK2 cells in NK cells **(B)** and in lymphocytes **(C)** from Young CMV-(n=4), Young CMV+(n=35), and Elder CMV+ (n=27) individuals.

**Figure S6**


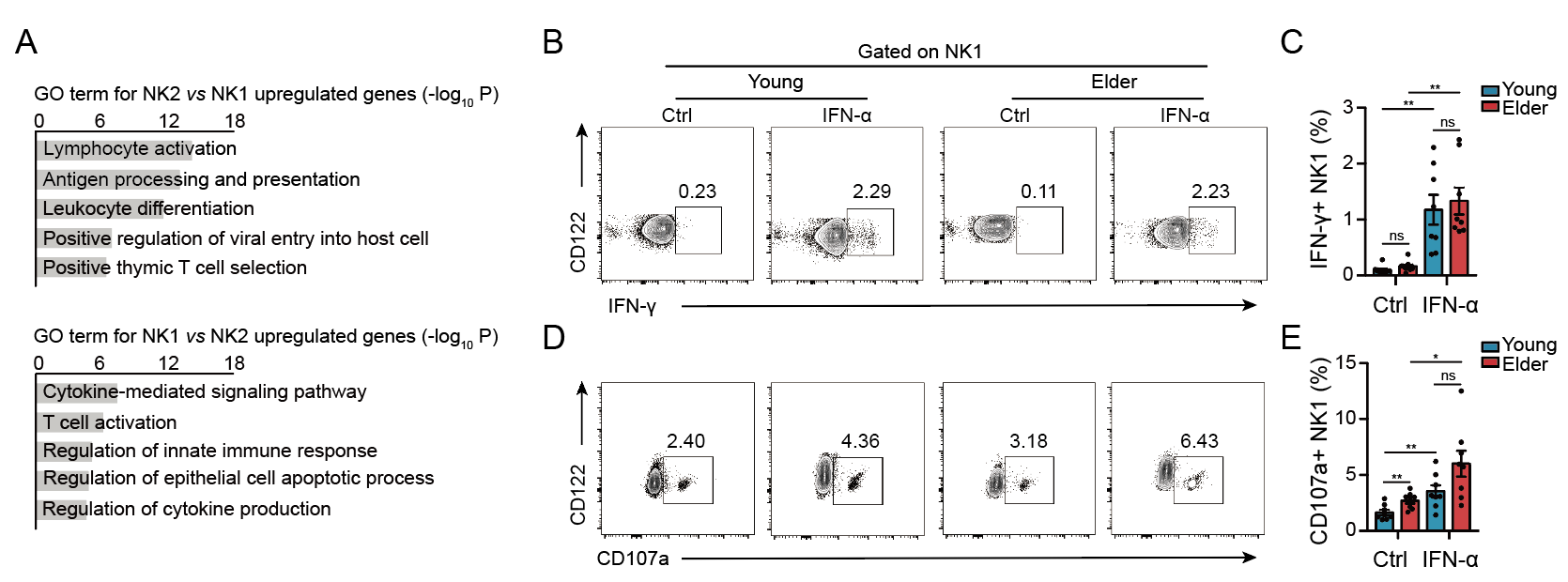


**Fig. S6 | NK2 cells in elderly individuals secrete elevated levels of IFN-γ and CD107a. A,** Gene ontology (GO) enrichment analysis of the differentially expressed genes of NK1 and NK2 cells from young and elderly individuals. **B, D,** Flow cytometry analysis of IFN-γ (**B**) and CD107a (**D**) expression in NK1 cells from young and elderly individuals with or without IFN-α stimulation *in vitro*. **C, E,** Bar plots displaying the frequencies of IFN-γ^+^ (**C**) and CD107a (**E**) in the NK1 subpopulation from young (n=8) and elderly (n=8) individuals with or without IFN-α stimulation *in vitro*. * *P* < 0.05; ** *P* < 0.01; ns, not significant. Student’s t-test.

**Figure S7**


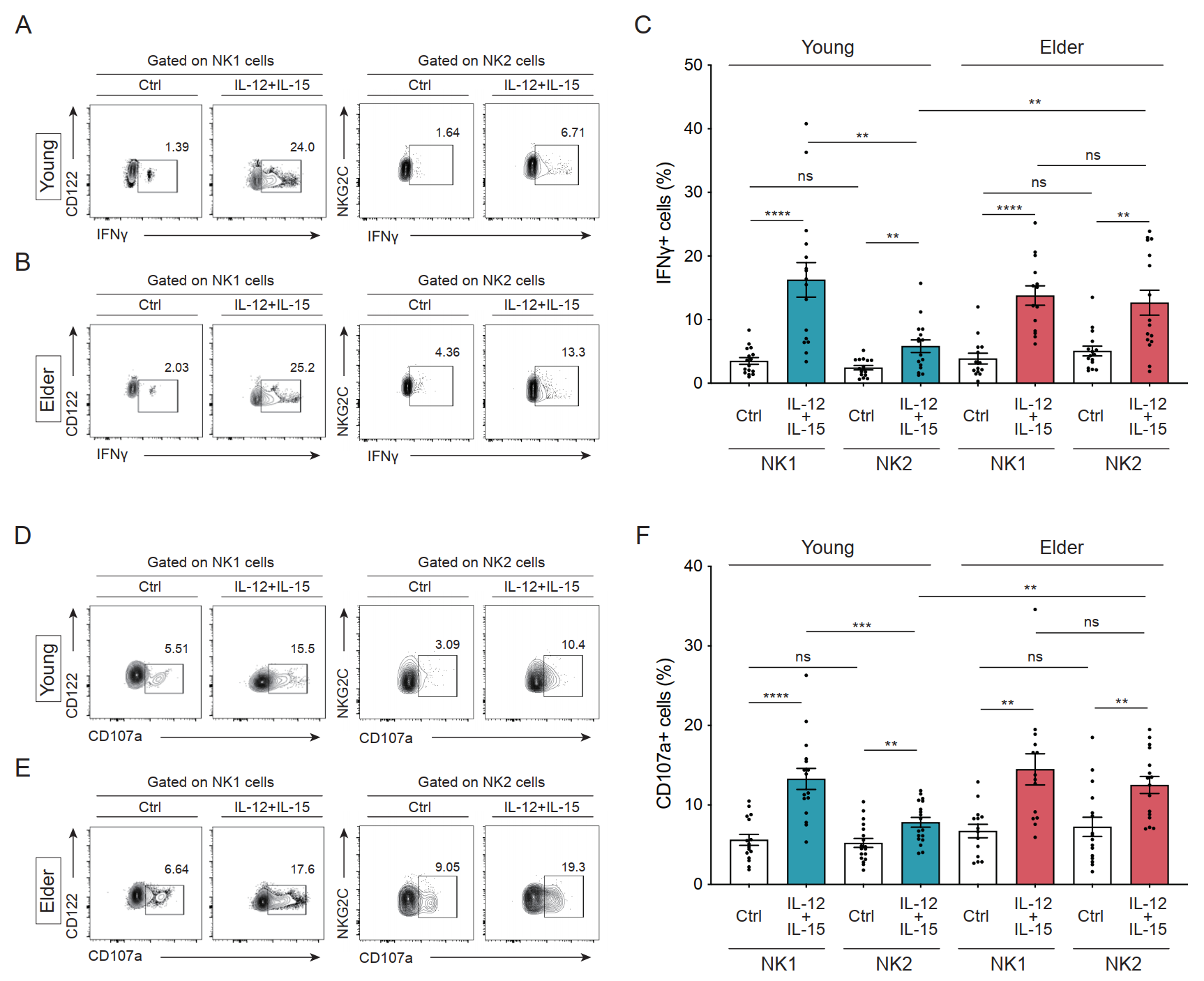


**Fig. S7 | Expression of IFN-γ and CD107a in NK1 cells and NK2 cells from young and elderly individuals.** **A, B,** Flow cytometry analysis of IFN-γ expression in NK1 cells (left panel) and in NK2 cells (right panel) from young (**A**) and elderly individuals (**B**) with or without IL-12+IL-15 co-stimulation *in vitro*. **C,** Bar plots displaying the frequencies of IFN-γ^+^ cells in the NK1 and NK2 subpopulation from young (n=16-18) and elderly individuals (n=14-16) with or without IL-12+IL-15 co-stimulation *in vitro*. **D, E,** Flow cytometry analysis of CD107a expression in NK1 cells (left panel) and in NK2 cells (right panel) from young (**D**) and elderly individuals (**E**) with or without IL-12+IL-15 co-stimulation *in vitro*. **F,** Bar plots displaying the frequencies of CD107a^+^ cells in the NK1 and NK2 subpopulation from young (n=16-18) and elderly individuals (n=14-16) with or without IL-12+IL-15 co-stimulation *in vitro*. ** *P* < 0.01; *** *P* < 0.001; **** *P* < 0.0001; ns, not significant. Student’s t-test.

**Figure S8**


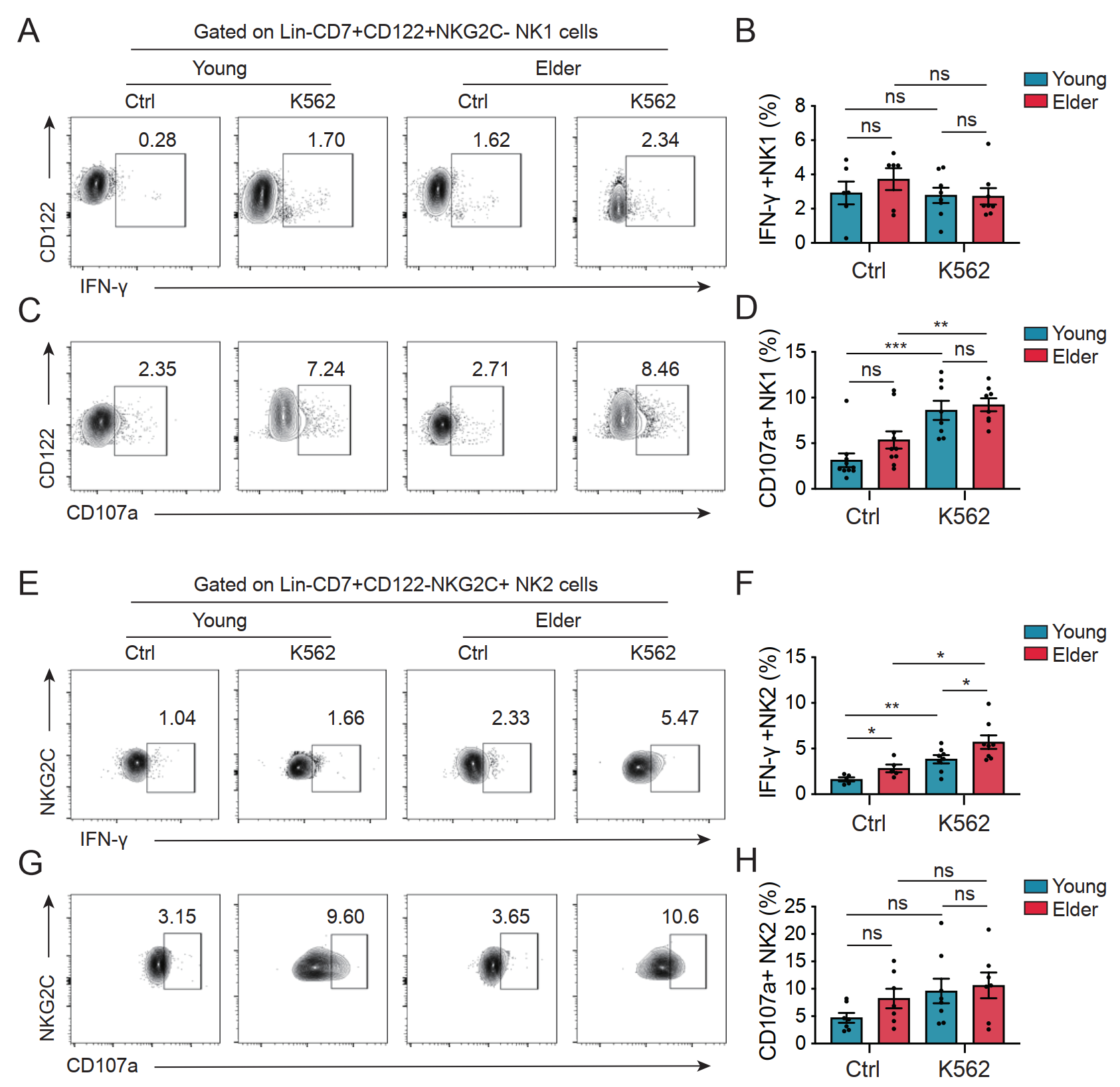


**Fig. S8 | Expression levels of IFN-γ and CD107a in Lin^-^CD7^+^CD122^+^NKG2C^-^ NK1 and in Lin^-^CD7^+^CD122^-^NKG2C^+^ NK2 cells from young and elderly individuals with or without K562 stimulation *in vitro*.** **A**, **E**, ﻿Flow cytometry analysis of IFN-γ expression in Lin^-^CD7^+^CD122^+^NKG2C^-^ NK1 cells (A) and in Lin^-^CD7^+^CD122^-^NKG2C^+^ NK2 cells (E) from young and elderly individuals with or without K562 stimulation *in vitro*. **B**, **F**, ﻿﻿Bar plots showing the expression of IFN-γ in Lin^-^CD7^+^CD122^+^NKG2C^-^ NK1 cells (B) and in Lin^-^CD7^+^CD122^-^NKG2C^+^ NK2 cells (F) from young (n=5-10) and elderly individuals (n=5-10) with or without K562 stimulation *in vitro*. **C**, **G**, ﻿Flow cytometry analysis of CD107a expression in Lin^-^CD7^+^CD122^+^NKG2C^-^ NK1 cells (C) and in Lin^-^CD7^+^CD122^-^NKG2C^+^ NK2 cells (G) from young and elderly individuals with or without K562 stimulation *in vitro*. **D**, **H**, ﻿﻿Bar plots showing the expression of CD107a expression in Lin^-^CD7^+^CD122^+^NKG2C^-^ NK1 cells (D) and in Lin^-^CD7^+^CD122^-^NKG2C^+^ NK2 cells (H) from young (n=5-10) and elderly individuals (n=5-10) with or without K562 stimulation *in vitro*. * *P* < 0.05; ** *P* < 0.01; *** *P* < 0.001; ns, not significant. Student’s t-test.

**Figure S9**


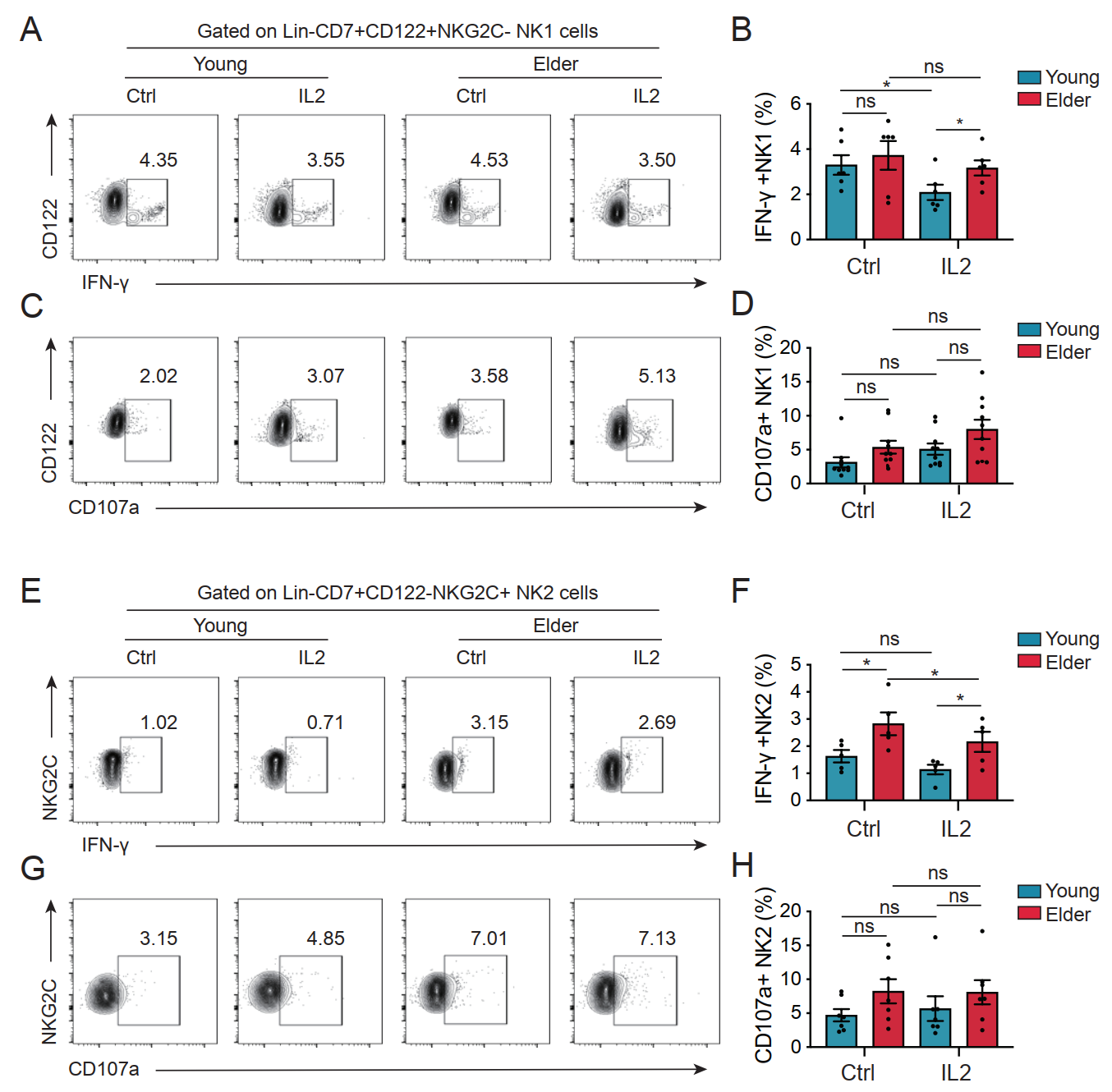


**Fig. S9 | Expression levels of IFN-γ and CD107a in Lin^-^CD7^+^CD122^+^NKG2C^-^ NK1 and in Lin^-^CD7^+^CD122^-^NKG2C^+^ NK2 cells from young and elderly individuals with or without IL-2 stimulation *in vitro*.** **A**, **E**, ﻿Flow cytometry analysis of IFN-γ expression in Lin^-^CD7^+^CD122^+^NKG2C^-^ NK1 cells (A) and in Lin^-^CD7^+^CD122^-^NKG2C^+^ NK2 cells (E) from young and elderly individuals with or without IL-2 stimulation *in vitro*. **B**, **F**, ﻿﻿Bar plots showing the expression of IFN-γ in Lin^-^CD7^+^CD122^+^NKG2C^-^ NK1 cells (B) and in Lin^-^CD7^+^CD122^-^NKG2C^+^ NK2 cells (F) from young (n=5-10) and elderly individuals (n=5-10) with or without IL-2 stimulation *in vitro*. **C**, **G**, ﻿Flow cytometry analysis of CD107a expression in Lin^-^CD7^+^CD122^+^NKG2C^-^ NK1 cells (C) and in Lin^-^CD7^+^CD122^-^NKG2C^+^ NK2 cells (G) from young and elderly individuals with or without IL-2 stimulation *in vitro*. **D**, **H**, ﻿﻿Bar plots showing the expression of CD107a expression in Lin^-^CD7^+^CD122^+^NKG2C^-^ NK1 cells (D) and in Lin^-^CD7^+^CD122^-^NKG2C^+^ NK2 cells (H) from young (n=5-10) and elderly individuals (n=5-10) with or without IL-2 stimulation *in vitro*. * *P* < 0.05; ns, not significant. Student’s t-test.

**Figure S10**


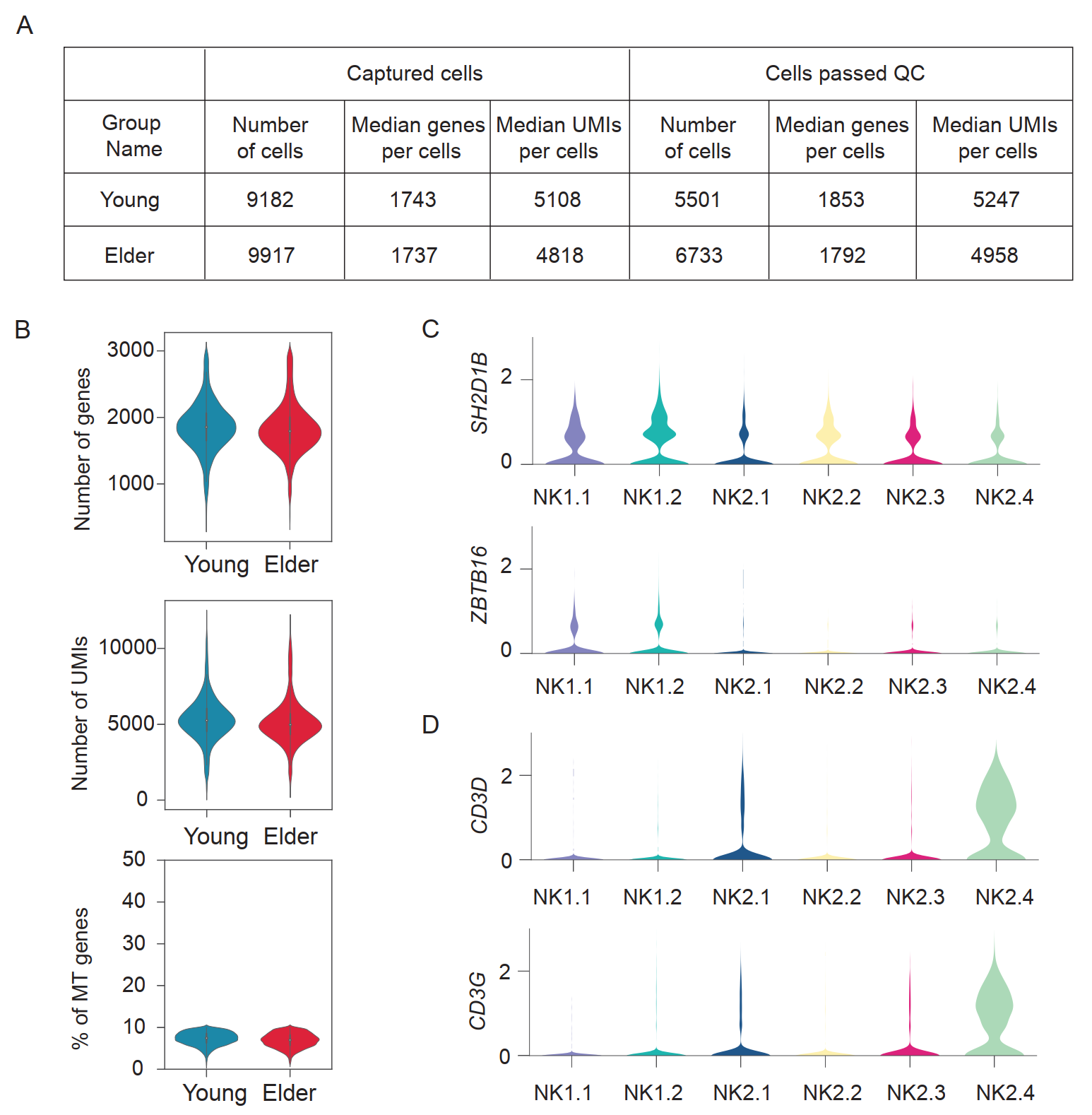


**Fig. S10 | Quality control of single-cell data for NK cells from young and elderly individuals.** **A,** Summary of captured cells, median genes per cell, median UMIs per cell, and the number of cells that passed quality control (QC) criteria for the single-cell data. **B**, Violin plots showing the gene number, UMI number, and percentage of mitochondrial RNA in single-cell data from young and elderly individuals. **C, D,** Violin plot showing the expression of the *SH2D1B*, *ZBTB16* **(C)**, *CD3D*, and *CD3G* **(D)** genes in NK1.1, NK1.2, NK2.1, NK2.2, NK2.3, and NK2.4 cells.

**Figure S11**


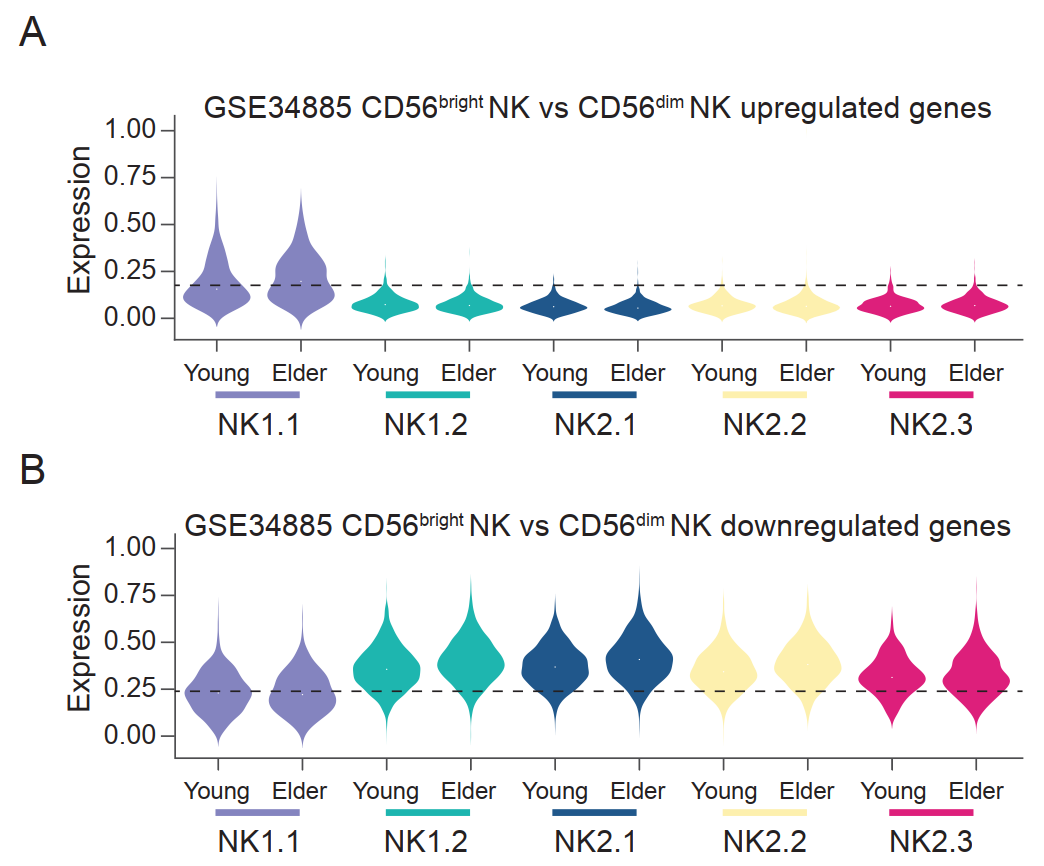


**Fig. S11 | Identification of NK cell subsets. A, B**, Violin plots showing the expression levels of upregulated (**A**) /downregulated (**B**) genes from a comparison of CD56^bright^ vs CD56^dim^ NK cells in NK1.1, NK1.2, NK2.1, NK2.2, and NK2.3 cells.

**Figure S12**


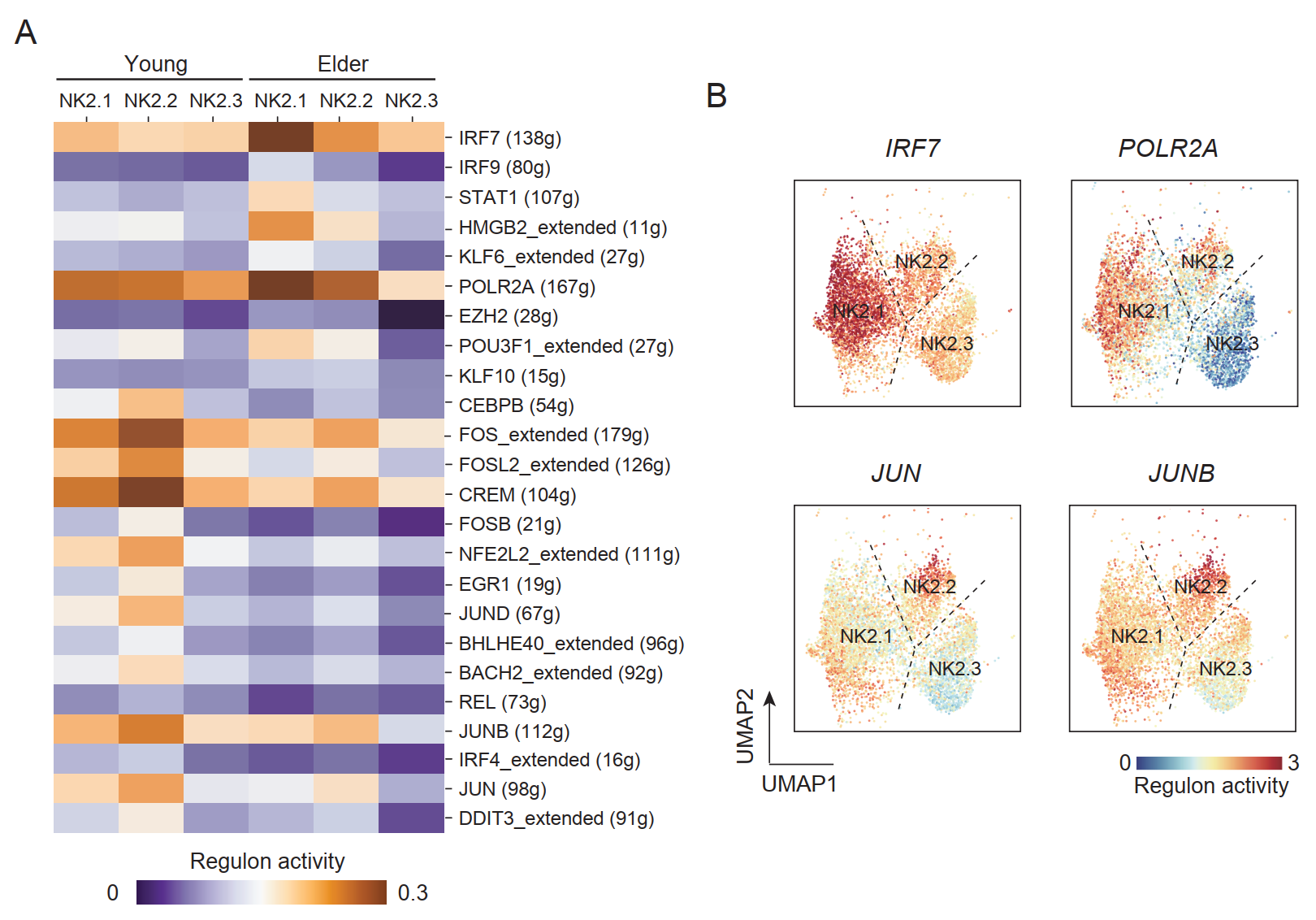


**Fig. S12 | Age-associated transcription factors of distinct NK cell subsets.** **A,** Heatmap of the AUC scores predicted by SCENIC for expression regulation by transcription factors (TFs) in NK2.1, NK2.2, and NK2.3 cells from young and elderly individuals. **B,** UMAP plots showing the AUC of the estimated regulon activity for *IRF7*, *POLR2A, JUN*, and *JUNB* in NK2.1, NK2.2, and NK2.3 cells.

**Figure S13**


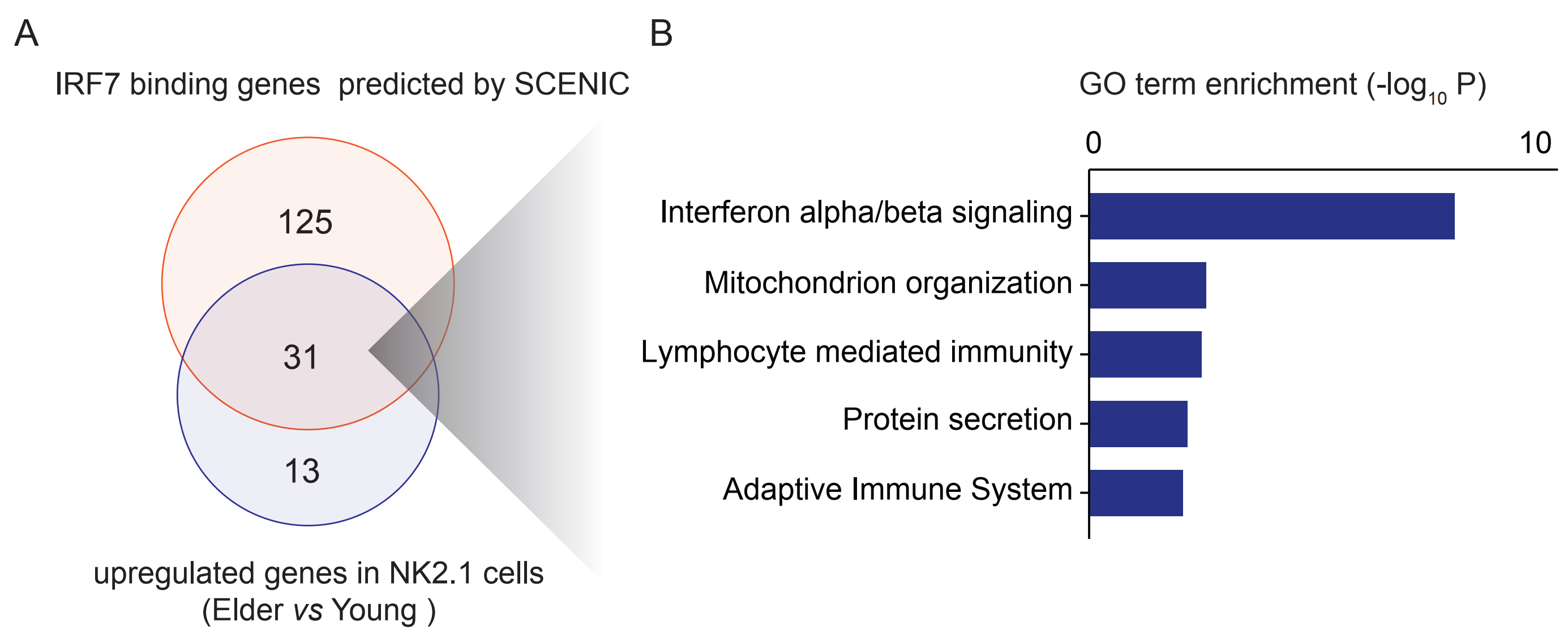


**Fig. S13 | Intersection of IRF7 binding genes predicted by SCENIC and upregulated genes in NK2.1 cells from a comparison of elderly vs. young individuals. A,** Venn diagram showing the intersection of IRF7 binding genes predicted by SCENIC and upregulated genes in NK2.1 cells from a comparison of elderly vs*.* young. **B,** GO enrichment analysis showing the functional annotations of genes in the intersection of IRF7 binding genes predicted by SCENIC and upregulated genes in NK2.1 cells from a comparison of elderly vs*.* young.

**Figure S14**


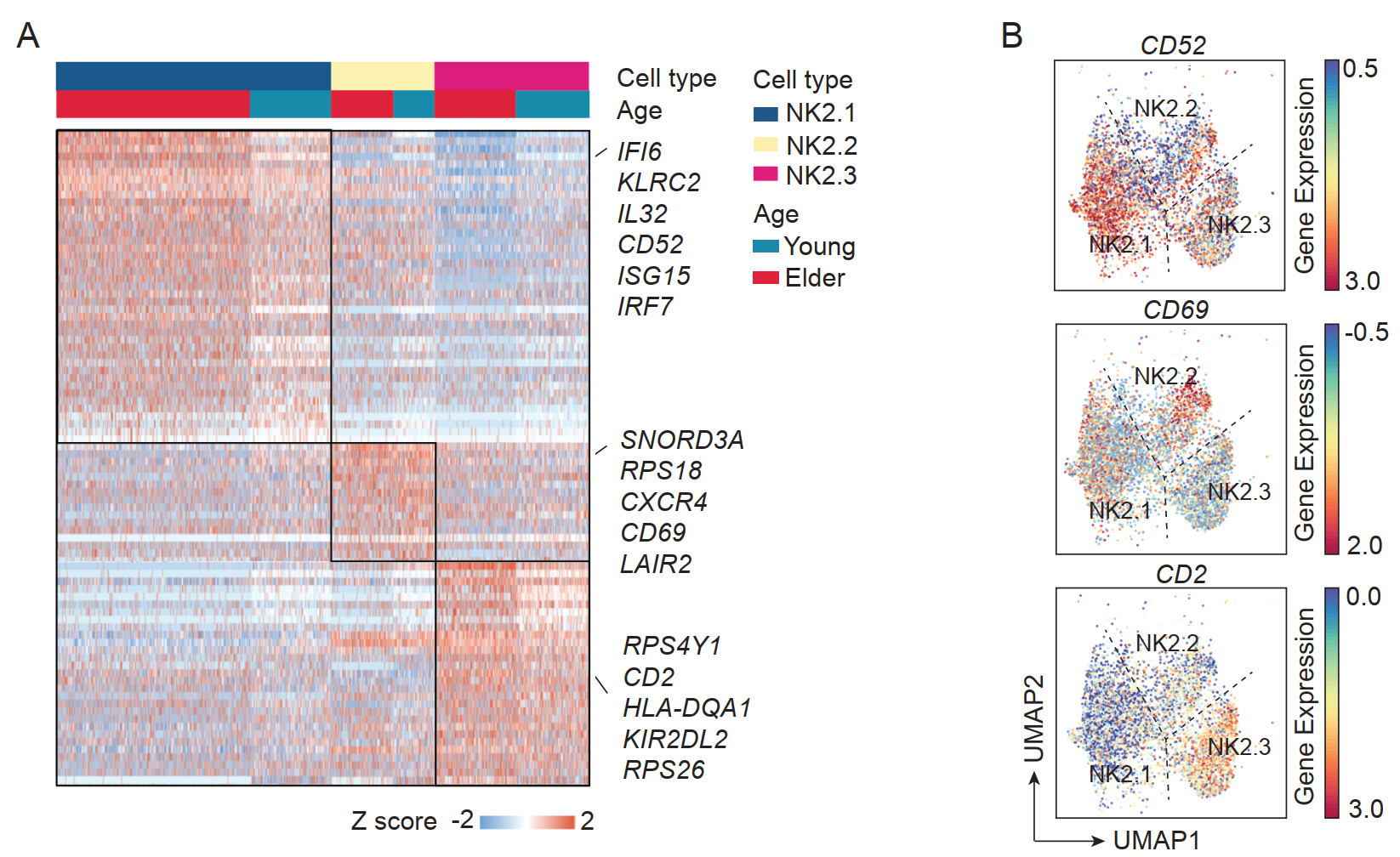


**Fig. S14 | Differentially expressed genes in NK2.1, NK2.2, and NK2.3 cells. A,** Heatmap showing the differentially expressed genes among NK1, NK2, and NK3 cells. **B**, UMAP graph showing the expression levels of *CD52*, *CD69*, and *CD2* in NK2.1, NK2.2, and NK2.3 cells.

**Figure S15**


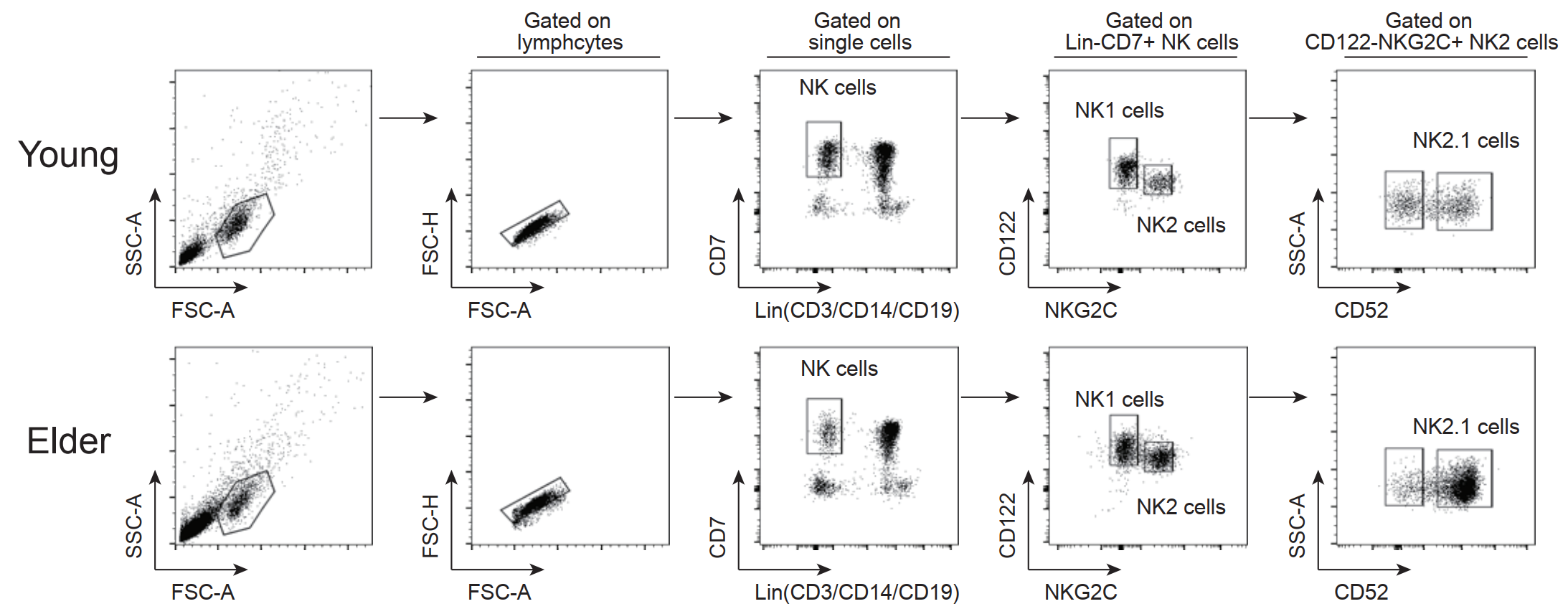


**Fig. S15 |** FACS gating strategy for Lin^-^CD7^+^CD122^-^NKG2C^+^ CD52^+^ NK2.1 cells from young and elderly individuals.

**Figure S16**


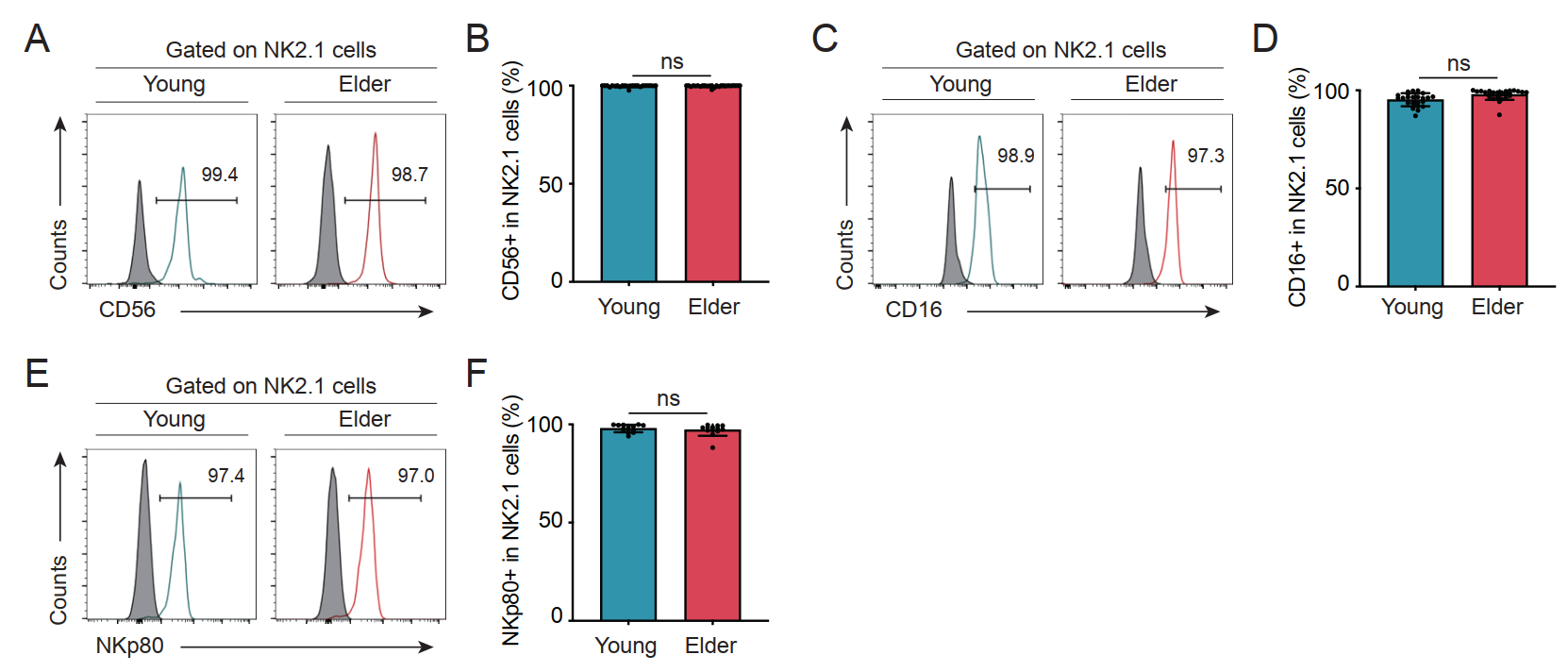


**Fig. S16 | Expression levels of NK-defining surface molecules (*i.e.*, CD56, CD16, and NKp80) in gated NK2.1 cells from young and elderly individuals. A**, **C**, **E**, FACS staining strategy for CD56 (A), CD16 (C), and NKp80 (E) expression in gated NK2.1 cells from young and elderly individuals. **B**, **D**, **F**, Bar plots showing the percentage of CD56 (B), CD16 (D), and NKp80 (F) expression in gated NK2.1 cells from young (n=14-23) and elderly individuals (n=13-25). ns, not significant. Student’s t-test.

**Figure S17**

**
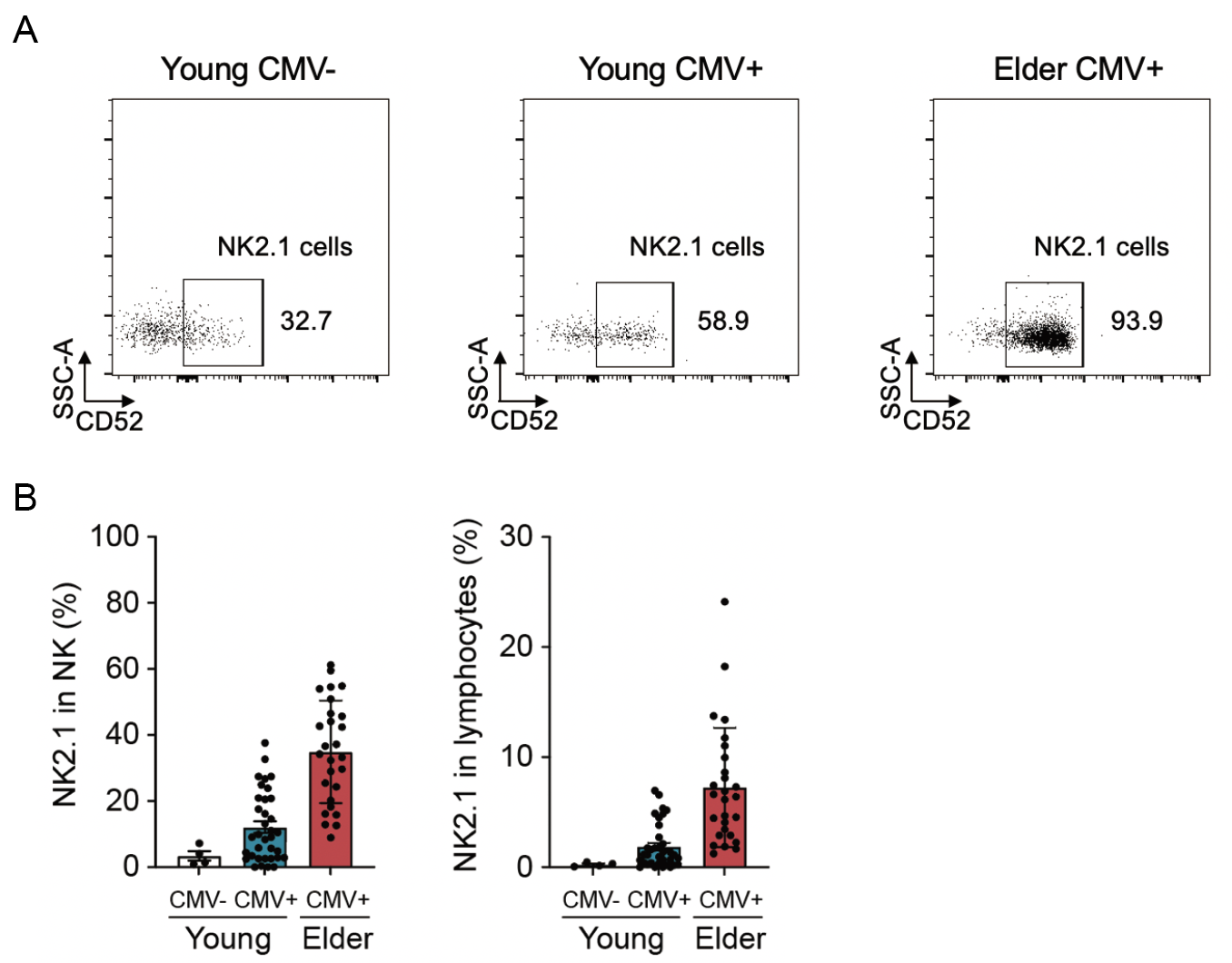
**

**Fig. S17 | CD52^+^NKG2C^+^CD122^low^ NK2.1 cells expand in CMV seropositive elderly individuals.** **A**, FACS staining strategy for NK2.1 cells (Lin^-^CD7^+^NKG2C^+^CD122^low^CD52^+^) from CMV seronegative young (Young CMV-), CMV seropositive young (Young CMV+), and CMV seropositive elderly (Elder CMV+) individuals. **B,** Bar plots showing the proportions of NK2.1 cells in NK cells (left) and in lymphocytes (right) from Young CMV-(n=4), Young CMV+(n=35), and Elder CMV+ (n=27) individuals.

**Figure S18**

**
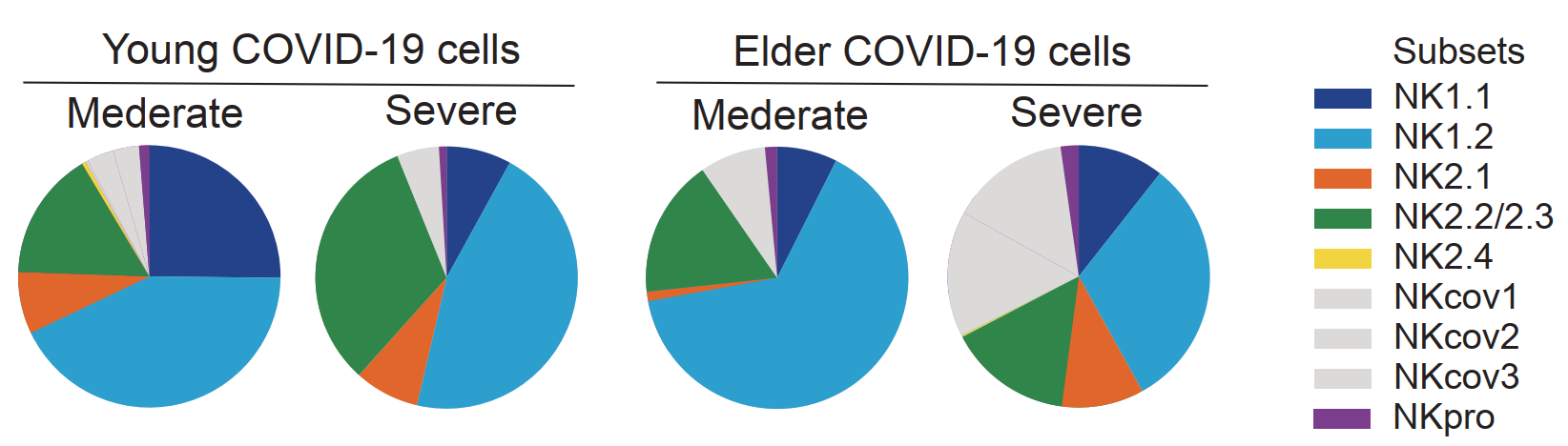
**

**Fig. S18 | NK cell subsets in COVID-19 patients.** Pie chart showing the proportions of the 9 NK subsets among NK cells from young (top) and elderly (bottom) COVID-19 patients.

**Figure S19**


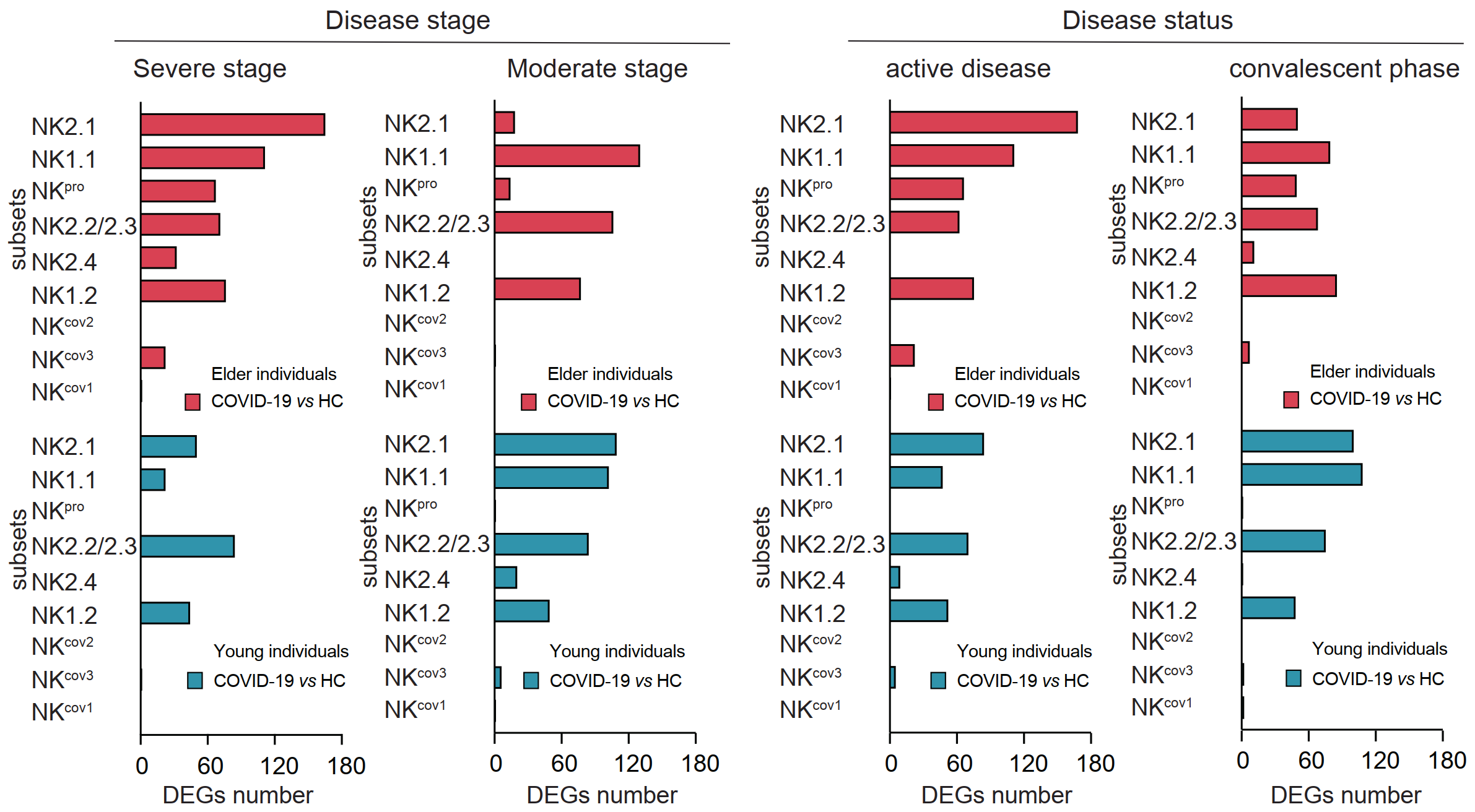


**Fig. S19 |** Histograms showing the number of DEGs for each NK cell subset in the elderly individuals (top) and the young individuals (bottom) between severe-stage COVID-19 patients and healthy controls (1^st^ column), moderate-stage COVID-19 patients and healthy controls (2^nd^ column), active-disease COVID-19 patients and healthy controls (3^rd^ column), and convalescent-phase COVID-19 patients and healthy controls (4^th^ column).

**Figure S20**

**
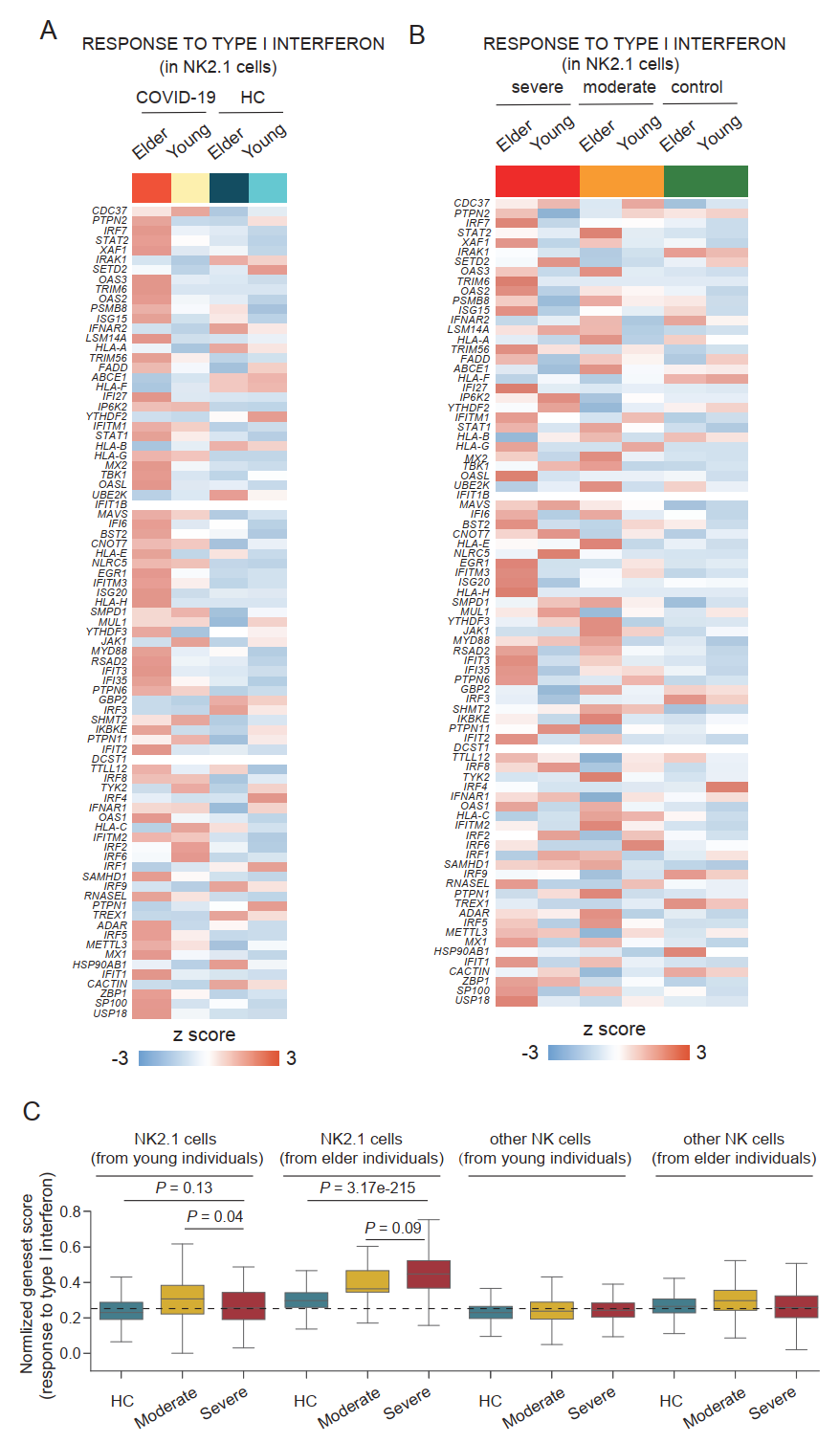
**

**Fig. S20 | Response to type I interferon in the NK2.1 cell subset positively correlates with disease progression and age. A,** Heatmap of the average expression levels of genes of the signalling pathway ‘response to type I interferon’ in NK2.1 cells from elderly COVID-19 patients, young COVID-19 patients, elderly healthy control individuals, and young healthy control individuals. **B,** Heatmap of the average expression of genes of the signalling pathway ‘response to type I interferon’ in NK2.1 cells from elderly COVID-19 patients at severe stage, young COVID-19 patients at severe stage, elderly COVID-19 patients at moderate stage, young COVID-19 patients at moderate stage, elderly healthy control individuals, and young healthy control individuals. **C,** Box plots of the normalized expression of genes involved in the signalling pathway ‘response to type I interferon’ in NK2.1 cells or other NK cells from healthy control individuals, moderate COVID-19 patients, and severe COVID-19 patients in young individuals (left) and elderly individuals (right). *P* values were obtained with Wilcoxon rank-sum tests.
